# Molecular mechanisms in liver repair and regeneration: from physiology to therapeutics

**DOI:** 10.1038/s41392-024-02104-8

**Published:** 2025-02-08

**Authors:** Xiao Ma, Tengda Huang, Xiangzheng Chen, Qian Li, Mingheng Liao, Li Fu, Jiwei Huang, Kefei Yuan, Zhen Wang, Yong Zeng

**Affiliations:** Division of Liver Surgery, Department of General Surgery and Laboratory of Liver Surgery, and State Key Laboratory of Biotherapy, West China Hospital, Sichuan University, Chengdu, Sichuan Province China

**Keywords:** Cell biology, Metabolic disorders, Reprogramming, Self-renewal

## Abstract

Liver repair and regeneration are crucial physiological responses to hepatic injury and are orchestrated through intricate cellular and molecular networks. This review systematically delineates advancements in the field, emphasizing the essential roles played by diverse liver cell types. Their coordinated actions, supported by complex crosstalk within the liver microenvironment, are pivotal to enhancing regenerative outcomes. Recent molecular investigations have elucidated key signaling pathways involved in liver injury and regeneration. Viewed through the lens of metabolic reprogramming, these pathways highlight how shifts in glucose, lipid, and amino acid metabolism support the cellular functions essential for liver repair and regeneration. An analysis of regenerative variability across pathological states reveals how disease conditions influence these dynamics, guiding the development of novel therapeutic strategies and advanced techniques to enhance liver repair and regeneration. Bridging laboratory findings with practical applications, recent clinical trials highlight the potential of optimizing liver regeneration strategies. These trials offer valuable insights into the effectiveness of novel therapies and underscore significant progress in translational research. In conclusion, this review intricately links molecular insights to therapeutic frontiers, systematically charting the trajectory from fundamental physiological mechanisms to innovative clinical applications in liver repair and regeneration.

## Introduction

On average, adult humans experience liver damage many times throughout their lifetime, especially under physiological conditions. The liver is a unique organ with a remarkable capacity for repair and regeneration, processes associated with survival and recovery after various forms of damage. Previous studies revealed that liver repair depends not only on the regeneration of isolated hepatocytes but also on various cellular interactions.^1^ This collaborative effort is crucial for the effective restoration of liver function and structure. Furthermore, the underlying molecular mechanisms that orchestrate these complex cellular interactions have been identified. These mechanisms involve key signaling pathways that determine not only cellular behavior but also the ability of the liver to rebuild.

Signaling pathways are complex networks of molecules that transmit signals from the external environment of the liver to initiate internal mechanisms, effectively guiding repair and regeneration processes. The intricacies of these pathways, including their activation, interaction, and regulation, are central to understanding how the liver responds to injury and initiates the repair process.^2^ Exploring the molecular mechanisms of metabolic reprogramming provides new insights into liver repair and regeneration. Research indicates that metabolic changes in cells are tightly interlinked with signaling pathways that regulate cell growth, survival, and function.^3,4^ The liver is not only the center of a complex signaling network but also the center of metabolic homeostasis. The liver is involved in regulating multiple metabolic processes, including glucose metabolism, lipid metabolism and amino acid metabolism.^5^ Dysfunction of metabolic homeostasis is associated with multiple diseases, highlighting the importance of the liver in maintaining the overall metabolic balance.^6^ There have been studies revealing that metabolites and metabolic enzymes serve as key factors that influence signaling networks by providing molecules that act as energy sources, substrates, co-factors, and signals themselves.^7–10^ Additionally, changes in cellular metabolism, induced by signals from various pathways, affect cell function and fate in response to different pathological conditions.^11,12^ Metabolic reprogramming supports the ability of the liver to adapt to its metabolic landscape in response to various types of damage, ensuring that sufficient resources and energy are available for cell proliferation and repair. Thus, a deeper understanding of the molecular mechanisms driving metabolic reprogramming could advance the development of therapeutic strategies.

Herein, we distinguish between classical signaling pathways and metabolic pathways by defining the former as those predominantly involving non-metabolic enzymes. This distinction allows for a comprehensive discussion of the latest advancements in the molecular mechanisms involved in liver repair and regeneration, particularly through the lens of complex signaling networks. This thorough analysis enriches our understanding of these processes and paves the way for innovative therapeutic interventions.

## Research milestones in liver repair and regeneration

**Fig. 1:**
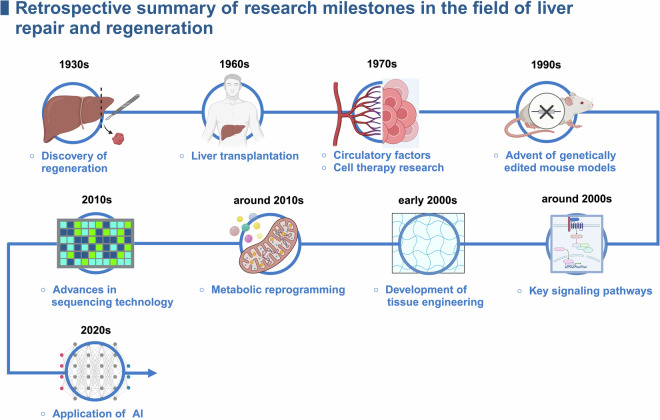
Retrospective summary of research milestones in the field of liver repair and regeneration. The linear timeline shows outstanding contributions to the field in different eras. AI, artificial intelligence. The figure was generated with BioRender (https://biorender.com)

Liver repair and regeneration have been fascinating and complex topics in biomedical research dating back centuries. Liver regeneration, recorded in myths and legends since ancient times, began receiving scientific attention in the early 20th century. The study of liver regeneration has progressed from merely observing the phenomenon to in-depth investigations of its molecular mechanisms. In recent decades, with rapid advances in molecular biology and genomics, we have achieved an unprecedented understanding of the cellular response and molecular mechanisms involved in liver repair and regeneration. These milestones not only demonstrate scientific and technological progress but also highlight groundbreaking discoveries that shape our current understanding of the signaling pathways related to repair and reflect our growing awareness of the complex functions of this vital organ in the human body **(**Fig. 1**)**.

### Discovery of the liver’s regenerative capacity

The earliest research on liver repair and regeneration began in the early 20th century. Two-thirds hepatectomy was successfully performed on rodents in the 1930s, and the remarkable ability of the liver to regenerate was recognized. Post-PH hypoglycemia can promote Cyclin D1 expression to induce early G1 progression.^13^ This finding highlights the physiological phenomenon of the liver repair capacity in response to injury. In the decades that followed, research focused on cellular morphology, illustrating how hepatocytes adapt their nuclear and chromosomal architectures to handle damage and proceed with repair and regeneration.^14–17^

### Liver transplantation: a clinical breakthrough shaping regeneration research

Liver transplantation represents a milestone in liver regeneration. The first successful human liver transplant was performed by Thomas E. Starzl in 1963.^18^ It represents not only represents a significant breakthrough in clinical medicine, providing new hope to patients with advanced liver disease, but also enriches our scientific understanding of liver biology, particularly the regenerative capacity. Liver transplantation enables direct observations of the liver repair process under extreme conditions, providing insights into the mechanisms and signaling pathways involved in regeneration. Additionally, liver transplantation provides valuable experimental models and clinical experience for researching new treatments for liver diseases and advancing regenerative medicine, thus fostering innovation and development in the treatment of individuals with liver conditions.

### Role of circulatory factors and cellular crosstalk in modulating liver regeneration

A series of circulatory factors were subsequently found to affect liver regeneration in the 1970s. Among these studies, the most famous is that of Starzl and colleagues, who reported that pancreas-derived humoral factors play crucial roles in liver regeneration.^19,20^ These investigations revealed that liver regeneration is a multistep process influenced by various factors.^21^ A key milestone in liver regeneration research was the subsequent discovery of the role of growth factors, such as hepatocyte growth factor (HGF), epidermal growth factor (EGF), and transforming growth factor α (TGF-α).^22^ Relevant studies have revealed the important role of changes in the microenvironment in the repair process. Subsequent studies revealed that non-parenchymal cells regulate liver regeneration.^23,24^ Meijer et al. reported that macrophages participate in initiating hepatocyte proliferation via growth factors and cytokines.^24^

While much research has focused on hepatocyte proliferation, the understanding of how necrotic liver lesions are resolved remains limited. A recent study revealed that monocyte-derived macrophages (MoMFs) are key, as they encapsulate necrotic areas and coordinate with hepatic stellate cells and SOX9^+^ hepatocytes through the JAG1/NOTCH2 signaling pathway and complement component C1q.^25^ This process not only clears necrotic debris but also advances liver repair, opening new avenues for therapeutic strategies in liver repair and regeneration.

### Progress in stem cell research for liver therapy

The first research on cell therapy dates back to 1976, when Najarian pioneered the transplantation of allogeneic hepatocytes into rats with congenital enzyme deficiency disease.^26^ Evidence for cell-mediated therapy subsequently attracted increasing amounts of attention. The discovery of liver stem cells or progenitor cells marked a significant advance, offering new avenues for therapy.^27^ Historically, the initial liver cell type recognized to possess stem/progenitor characteristics was rat oval cells located in the canals of Hering.^28^ Subsequent lineage tracing methods led to the revision of the early understanding of oval cells and indicated that hepatocytes are able to renew to maintain the liver mass in mice.^29–31^ Moreover, women who receive bone marrow transplants from male donors have shown the presence of the Y chromosome in their hepatocytes. This finding indicates that hepatocytes can originate from the bone marrow via transdifferentiation.^32^ A consensus on a definitive liver stem/progenitor cell is still lacking. In the future, advances in technology may contribute to revealing cellular plasticity in the context of different liver diseases.^33,34^

### The role of genetically edited mouse models in unraveling liver regeneration mechanisms

Before the advent of genetically edited animal models, a conclusive demonstration of the functions and mechanisms of these growth factors was difficult, as researchers lacked the tools to specifically enhance or inhibit them in vivo. The 1991 study that employed the transgenic albumin-urokinase type plasminogen activator (Alb-uPA) mouse model is recognized as one of the early landmark studies in which genetic manipulation was used to investigate liver regeneration.^35^ In the Alb-uPA mouse model, uPA, which is controlled by an albumin promoter, converts plasminogen into plasmin inside hepatocytes, causing proteolysis and apoptosis. This process leads to continuous liver damage and eventual failure.^35^

### Key molecular insights from advances in genetics

Advances in molecular biology and genetics around 2000 led to an in-depth understanding of the mechanisms controlling liver regeneration. Key genes and signaling pathways, such as the Wnt/β-catenin, MET, and signal transducer and activator of transcription 3 (STAT3) pathways, have been identified.^33^ During this period, another important advance was revealing the importance of posttranslational modifications.^2,36^

### Regenerative medicine and tissue engineering

In the early 2000s, researchers have focused on regenerative medicine approaches, including the use of bioartificial liver devices and liver tissue engineering. These approaches involve the use of stem cells and biomaterials to create a supportive environment for cellular growth and differentiation.^37^

### Metabolic reprogramming driving repair and regeneration

Since around 2010s, the role of metabolic reprogramming in liver regeneration has become a focal point of research.^38–41^ This process adjusts cellular metabolism to meet the repair needs of the liver, shifting key pathways such as glycolysis, fatty acid metabolism, and oxidative phosphorylation.^38,42^ These adaptations provide the energy, materials, and signals necessary for hepatocyte proliferation and tissue repair.^43^ Advances in metabolomics have deepened our understanding of how these metabolic changes facilitate liver regeneration, providing new insights into potential therapeutic targets for enhancing liver repair.

### Advances in sequencing technology unlocking cellular insights in liver research

In recent years, single-cell sequencing (scRNA-seq) technology has opened new possibilities for obtaining a more granular understanding of cellular heterogeneity and cell-specific gene expression.^44^ The introduction of third-generation sequencing technologies, around 2010, such as single-molecule real-time (SMRT) sequencing and nanopore sequencing, has further accelerated these advancements. These technologies provide longer read lengths, enhanced accuracy, and the ability to sequence individual molecules directly, thereby facilitating more detailed and comprehensive analyses of single-cell transcriptomes. Moreover, spatial transcriptomics provides spatial data for understanding how liver cells organize and interact spatially to promote repair and regeneration in different regions of the liver.^45^ In particular, ProTracer is a technology that tracks cell fate. It is able to provide dynamic information on cellular changes during liver regeneration.^46^ This information is crucial for understanding the self-repair mechanisms of the liver and developing new therapies for liver disease.

### Potential of artificial intelligence (AI) in advancing liver research and therapy

With the breakthrough of large-scale language models, AI began to experience exponential growth in the field of liver disease research around 2020. The application of AI in liver repair and regeneration is still in its early stages, but it has great potential to improve our understanding of molecular mechanisms and optimize treatment strategies for liver repair and regeneration.^47,48^

AI is able to process and analyze vast amounts of biomedical data, such as genomic, proteomic, and clinical data. Through advanced data analysis, AI is capable of identifying key molecular patterns and biomarkers of liver disease, leading to a deeper understanding of pathological molecular mechanisms.^49,50^ AI is able to create models that can be used to predict disease progression, the treatment response, and clinical outcomes.^51^ AI technology contributes to accelerating new drug discovery, helping to optimize potential drug candidates.^52^ With continuous advancements in AI, future applications in the field of liver disease will be more extensive and in greater depth.

## Cellular response in liver repair and regeneration

**Fig. 2:**
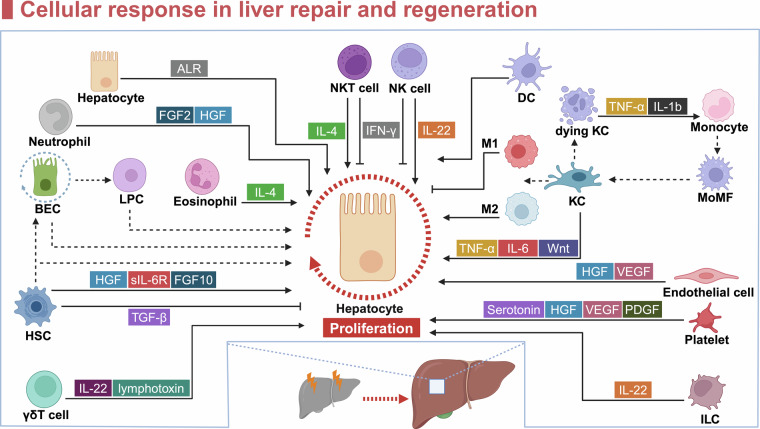
Roles of liver cells during liver repair and regeneration. Different cell subsets regulate the proliferation of hepatocytes by secreting cytokines and chemokines. The arrows indicate a stimulatory effect on proliferation, and the T-arrows indicate an inhibitory effect. The dotted lines indicate dedifferentiation, differentiation or transdifferentiation. The circular dotted line indicates renewal. HSC hepatic stellate cell, LPC liver progenitor cell, BEC biliary epithelial cell, NKT natural killer T, DC dendritic cell, KC kupffer cell, MoMF monocyte-derived macrophage, ILC innate lymphoid cell, ALR, augmenter of liver regeneration, FGF fibroblast growth factor, HGF hepatocyte growth factor, IL interleukin, sIL-6R, soluble IL-6R, TGF-β transforming growth factor β, IFN-γ interferon-gamma, TNF-α tumor necrosis factor α, VEGF vascular endothelial growth factor, PDGF platelet-derived growth factor. The figure was generated with BioRender (https://biorender.com)

The liver comprises a complex microenvironment enriched with various cell types, including parenchymal and non-parenchymal cells. These cells are instrumental in regulating key processes such as proliferation and differentiation, and they play pivotal roles in restoring liver function after injury (Fig. 2). Investigating how these cells interact and signal during the recovery phase can enhance our understanding of the molecular mechanisms of the liver and aid in the development of targeted therapeutic strategies.

### Biliary epithelial cells

Biliary epithelial cells (BECs), which are differentiated epithelial cells, are non-parenchymal cells that regulate bile excretion and homeostasis. Lineage tracing studies have revealed that hepatocytes typically replenish lost liver tissue through self-replication. However, when the extent of hepatocyte damage exceeds their capacity for self-replication, other liver cells are activated to undergo dedifferentiation or transdifferentiation, thereby compensating for the lost tissue.^28,53–56^ Activated BECs can acquire hepatocyte features to support liver regeneration via cellular reprogramming.^57^ The plasticity of BECs is considered a characteristic of stem cells. Studies on the transdifferentiation of BECs have generated substantially divergent opinions and controversies. Several studies have shown that BEC plasticity, which includes the dedifferentiation of BECs into bipotential progenitor cells and the subsequent transformation of progenitor cells into hepatocytes, is essential for liver regeneration.^53,58–61^ However, other studies have suggested that BECs, as facultative stem cells, directly transdifferentiate into hepatocytes in injured livers.^55,56,62^

During liver injury, cells expressing BEC markers proliferate from the periportal area into the adjacent parenchyma. These proliferating cells, which are part of the ductular response (DR), also express ductular markers such as Sox9.^63,64^ DR activation is also found in humans with liver disease. Patients with acute liver failure exhibit impaired hepatocyte proliferation, which is accompanied by an intense DR.^65^ Moreover, BECs from cirrhotic patients with autoimmune or viral hepatitis express the hepatocyte marker HNF4α.^56^

Mechanistically, the Rngtt/mammalian target of rapamycin complex 1 (mTORC1)/dnmt1 axis has been shown to be essential for the dedifferentiation of BECs to liver progenitor cells (LPCs).^66,67^ Farnesoid X receptor (FXR), Wnt, and bone morphogenetic protein have also been shown to contribute to LPC differentiation into hepatocytes.^68–70^ Recent studies have explored the molecular mechanisms underlying the phenomenon of cell fate conversion by combining scRNA-seq and dual recombinase-mediated lineage tracing. The suppression of Notch signaling facilitates the transformation of BECs to LPCs and the subsequent activation of the Wnt signaling-mediated LPC-to-hepatocyte conversion.^53^ Future research should address the identification of new accurate markers to trace cell fate decisions under different injury conditions.

### Endothelial cells

Liver endothelial cells form a heterogeneous population essential for liver function and regeneration.^71,72^ Among them, liver sinusoidal endothelial cells (LSECs), which line the liver sinusoids, are specialized for facilitating molecular exchange, immune surveillance, and supporting regenerative processes.^73^ LSECs cooperate with parenchymal and non-parenchymal cells to induce hepatocyte proliferation.^72,74^

Nitric oxide (NO) secreted by LSECs regulates the sensitivity of hepatocytes to HGF in response to increased shear stress following resection.^75^ Besides, LSECs further facilitate liver regeneration by increasing the expression of uPA, which activates HGF in the extracellular matrix (ECM).^76^ LSECs also directly secrete HGF and Wnt2 to promote Id1-dependent hepatocyte mitosis.^77^ Although the relative contributions of HGF among various secretory cells remains unresolved, a deficiency of specific HGFs in LSECs results in impaired liver regeneration in adult mice following partial hepatectomy (PHx). These defects cannot be compensated by HGF produced by other cells.^75,76^ Building on this, LSECs produce vascular endothelial growth factor (VEGF), which acts as a key regulator by promoting the proliferation of both hepatocytes and LSECs. VEGF further stimulates the release of HGF from LSECs via VEGF/VEGFR2 signaling, reinforcing the regenerative process.^78–81^

Relative hypoxia occurs along with increased cell division of hepatocytes during the inductive phase. A hypoxic environment causes hepatocytes and HSCs to release VEGF, which stimulates LSEC proliferation and remodels the sinusoidal network.^82,83^ During the angiogenic phase, increased expression of angiopoietin-2 stimulates LSEC proliferation in an autocrine manner.^84^

### Hepatic stellate cells

HSCs are indigenous mesenchymal cells situated in the space of Disse. HSCs account for 8% of all cells and store up to 70–95% of all retinoid lipids (vitamin A) in the homeostatic liver.^85^

Several signaling molecules produced by HSCs contribute to hepatocyte proliferation. One of the most critical molecules is HGF. A large amount of HGF stored in the ECM is rapidly activated in a heterodimeric form after PHx. These molecules bind to MET on hepatocytes via a paracrine pathway and through the peripheral blood circulation. HGF heterodimers subsequently activate several downstream signaling pathways to drive the G1-S cell cycle transition.^86^ Fibroblast growth factor (FGF) 10 overexpression induced by HSCs also affects hepatocytes to drive hepatocyte proliferation during ischemia‒reperfusion injury (IRI).^87^ HSCs are mainly stimulated to produce hepatocyte growth factor by interleukin-6 (IL-6) trans-signaling rather than through IL-6 signaling following PHx, which indicates the crucial role of soluble IL-6 receptor (sIL-6R) in liver repair.^88^ Kimura et al. reported that one heterogeneous HSC cluster expressing the collagen gene is involved in restoring the liver mass via the induction of hepatocyte hypertrophy.^89^ In the associative liver partition and portal vein ligation for staged hepatectomy (ALLPS) model, Indian Hedgehog protein from HSCs is instrumental in promoting the renewal of liver parenchymal cells.^90^ A recent study revealed that senescent HSCs in young mice following PHx promote liver repair through multiple signaling pathways induced by the expression of the senescence-associated secretory phenotype.^91^ These findings contrast with the findings that senescent HSCs have a reduced ability to induce regeneration in aged rat livers.^92^ The complex role of HSCs in liver regeneration may depend on different contexts and intercellular crosstalk, which requires further investigation.

HSCs are also involved in the termination of liver regeneration. HSCs contribute to hepatic ECM reconstitution to isolate hepatocytes from growth factors during the termination of liver regeneration, which induces proliferating hepatocytes to exit the cell cycle.^93^ TGF-β secreted by HSCs inhibits the regenerative response of hepatocyte proliferation in the termination stage.^85,94^

Interestingly, HSCs have the potential to replenish the liver mass by differentiating into hepatocytes and bile duct cells under specific conditions.^95^ The potential mechanism involves the transdifferentiation of HSCs via the Hippo/Yes-associated protein (YAP) pathway.^96^

### Macrophages

Among the non-parenchymal cells involved in liver regeneration, the role of macrophages has been well highlighted in clinical patients and mouse models.^97^ Macrophages constitute the most abundant proportion of immune cells involved in homeostasis, representing approximately 20% of non-parenchymal cells.^98^ In fact, macrophages are heterogeneous and derive from different ontological origins during liver damage: resident yolk sac-derived macrophages, also known as Kupffer cells (KCs), and circulating bone marrow MoMFs. KCs are identified primarily as CD45^+^F4/80^high^CD11b^int^CLEC4F^+^Timd4^+^ cells, and MoMFs are characterized by the CD45^+^F4/80^int^CD11b^high^ expression. Liver regeneration is severely impaired when macrophages are depleted in response to acute liver injury, such as PHx, acetaminophen overdose, acute carbon tetrachloride (CCl_4_) or chronic liver injury.^99–102^

Previous studies have shown that macrophages provide the most important factors for hepatocyte proliferation by producing cytokines, including tumor necrosis factor α (TNF-α) and IL-6, after PHx.^1,103–105^ Once liver damage occurs, activated KCs begin to produce TNF-α via Toll-like receptor (TLR)/MyD88-mediated pathways, which triggers the expression of immediate early genes involved in switching hepatocytes from a quiescent state to a dividing state.^106^ Subsequently, TNF-α operates in an autocrine manner, further activating NF-κB. This activation of NF-κB reciprocally promotes the release of TNF-α and IL-6.^86,107,108^ Binding of IL-6 to hepatocyte receptors initiates the STAT3 signaling pathway. STAT3 dimers subsequently move to the nucleus and modulate cyclin D1 expression, influencing the G1/S transition in hepatocytes. Recent studies have revealed that the penta-span transmembrane glycoprotein Prom1 confines glycoprotein 130 (gp130) to lipid rafts and subsequently facilitates the activation of the IL6-gp130-STAT3 signaling pathway during liver regeneration.^109,110^ The activation of STAT3 also induces the expression of antiapoptotic genes such as Bcl-xL, B-cell lymphoma-2, and FLICE inhibitory proteins, ensuring the survival of hepatocytes during regeneration.^111^ Beyond its direct effect on hepatocytes, IL-6 can also indirectly promote liver regeneration by stimulating HSCs to secrete HGF, which mediates mitotic activation and recovery of the residual liver with other extrahepatic factors.^88^

KCs are a significant source of Wnt proteins, which stimulate β-catenin in hepatocytes in a paracrine manner following PHx.^112,113^ In fact, the stimulation of macrophages with TNF-α was sufficient to induce the synthesis of Wnt proteins, which are required for the activation of β-catenin and subsequent hepatocyte proliferation.^114^

In response to various stimuli, macrophages can differentiate into two unique subsets, referred to as M1 (proinflammatory) or M2 (anti-inflammatory) macrophages.^115^ M1 macrophages mediate excessive inflammatory responses, cytotoxicity and tissue damage by producing proinflammatory cytokines and chemokines,^116^ whereas M2 macrophages aid in reducing inflammation, tissue restoration, and cellular proliferation through the secretion of anti-inflammatory cytokines such as IL-10 and IL-4.^117^ Thus, the extent of inflammation and repair in the liver is largely influenced by the equilibrium between M1 and M2 macrophage polarization. Elchaninov et al. reported that an increase in the number of M2 macrophages may be associated with liver regeneration after PHx. An M2-MoMF infusion increases resident macrophage polarization, attenuates posthepatectomy liver dysfunction and promotes hepatocyte proliferation,^118^ whereas the suppression of hepatocyte apoptosis and aggravation of liver dysfunction are observed after an M1 infusion. Recent research has indicated that chemokines play a role in the polarization of macrophages.^119^ C-C motif chemokine ligand 5 (CCL5) deficiency or inhibition enhances liver repair by inducing M2 polarization and suppressing M1 polarization. Mechanistically, the blockade of CCL5 increases the production of HGF from reparative macrophages via the C‐C motif chemokine receptor CCR1- and CCR5‐mediated forkhead box protein O3 (FOXO3) pathways.^119,120^ However, liver regeneration is compromised by increased M2 macrophage polarization following extended hepatectomy, which is distinct from the traditional 70% PHx model. This distinct phenomenon may involve macrophages inhibiting IL-6 secretion by secreting PD-1.^121^

Recent findings indicate that the expression of flagellin, a subunit protein of the bacterial flagellum, increases in the liver and serum following PHx. The expression of its main receptor, TLR5, is also markedly upregulated. TLR5 is expressed mainly on KCs, recruited macrophages and hepatic neutrophils.^122,123^ Disruption of TLR5 signaling is associated with decreased proinflammatory cytokine production via the TLR/MyD88/NF-κB pathways. TLR5 activation also contributes to transient lipid accumulation, which is essential for physiological liver regeneration. These results suggest that the bacterial flagellin content may influence liver regeneration by regulating immunity and metabolism.^124^

Interestingly, KC death has been suggested to act altruistically to facilitate the recruitment of effector cells during inflammation. The release of TNF-a and IL-1b from dying KCs results in monocyte recruitment to the liver.^125^ Surviving hepatocytes around areas of necrosis trigger KC apoptosis by increasing the expression of CXCR4 on redundant KCs following acute liver injury, which could influence liver regeneration.^126^

While the roles of traditional macrophage subsets like KCs in liver regeneration have been well-documented, recent research has begun to shed light on the diverse and dynamic nature of the macrophage pool, particularly how it evolves from birth through repeated injury and regeneration cycles. The macrophage pool at birth is rather different from that when adult humans suffer repeated injury and regeneration cycles throughout their lifetime.^97,127^ The underlying mechanisms involved in macrophage replenishment during different pathophysiological processes are not identical. Newly identified macrophages also play potential roles in liver regeneration. Wang et al. reported that a group of F4/80^hi^GATA6^+^ macrophages have the potential to be mobilized from the peritoneal cavity to the liver, where they can utilize their significant regenerative ability to promote liver regeneration.^128^ Among these new subgroups, hemorrhage-activated macrophages (Mhems) play crucial roles in clearing the bloodstream of aged red blood cells by engulfing erythrocyte remnants and hemoglobin deposits. What sets Mhems apart from other macrophage subsets is their elevated HO-1 activity. However, the transfusion of stored red blood cells plays a negative role in the regenerative process through increased Mhem activation following PHx.^129^

### T cells

T cells can be categorized into two groups, αβ and γδ T cells, which are distinguished by their unique T-cell receptors (TCRs) and account for less than 10% of non-parenchymal T cells.^98^ γδT cells, which comprise an estimated 15%–25% of liver T cells, also function as either protective or harmful immune cells in the context of liver diseases. Increasing evidence reveals that T cells play important roles in regulating liver regeneration.^98^ In fact, an increase in the number of T cells (including both αβ T cells and γδ T cells) originating from extrahepatic recruitment has been noted in the liver following PHx.^130^ Studies have shown that mice lacking almost all T cells exhibit a 75% mortality rate after PHx and that mice lacking only γδT cells exhibit impaired liver regeneration.^131,132^ TCRβ deficiency leads to an increase in hepatocyte apoptosis and significant inhibition of hepatocyte replication, and αβT cell deficiency induces a compromised mechanism to increase the number of γδT cells involved in the liver regeneration process.^130^ Furthermore, γδT cells can directly induce hepatocyte proliferation via the production of IL-22 in a Dectin-1-dependent manner.^132^. Transgenic mice overexpressing IL-22 exhibit accelerated liver regeneration after PHx.^133^ Lymphotoxin produced by T cells increases IL-6 production by stimulating the lymphotoxin β receptor on hepatocytes.^131,134^ CD8^+^ T cells can also induce the proliferation of LPCs in the process of liver repair in models of chronic liver injury.^135^

As a subset of T cells, natural killer T (NKT) cells account for the greatest percentage of total lymphocytes in the liver.^136^ NKT cells are a highly heterogeneous subpopulation that expresses both T and NK cell surface markers.^137^ Although an increase in NKT cells has been observed after liver regeneration, their functions vary under different disease contexts. NKT cells negatively regulate liver regeneration via interferon-gamma (IFN-γ)-induced hepatocyte cell cycle arrest in HBV-Tg mice.^138^ In the setting of ampicillin-sensitive commensal bacteria depletion, IL-12-induced NKT cell overactivation impairs liver regeneration through increased IFN-γ production.^139,140^ The inhibition of liver regeneration by the IFN-γ/STAT1 pathway has been widely recognized.^141,142^ Other studies have shown that the direct cytotoxic effect of NKT cells on hepatocytes induces apoptosis to inhibit regeneration following PHx.^143,144^ The lipid antigen α-GalCer is known to stimulate NKT cells. Yin et al. reported that α-GalCer administration activates NKT cells to produce IFN-γ and impede liver regeneration.^145^ In contrast, Nakashima et al. reported that α-GalCer treatment activates NKT cells to promote liver regeneration via the TNF and Fas/Fas ligand-mediated pathways.^146^ This paradoxical effect of NKT cells on liver regeneration may depend on the status of activation, the mouse strain, or the research environment.^132,145,147^

NKT cells also regulate liver regeneration via a regulatory feedback loop involving the cleavage of C3 and C5 to produce C3a and C5a. Complement activation induces the recruitment of NKT cells and the subsequent production of IL-4 following PHx. Secreted IL-4 further promotes complement activation via increased IgM deposition.^148^

### Eosinophils

Eosinophils represent a unique cell type in the innate immune system and, in early studies, were shown to be involved in host responses, immunity and allergic inflammation.^149^ However, recent studies have revealed that the accumulation and activation of eosinophils play crucial roles in liver regeneration. The infiltration of eosinophils increases and contributes to liver regeneration following PHx. In fact, inhibition of the transcription of eotaxin, a special chemokine that primarily attracts eosinophils, subsequently impedes the recruitment of eosinophils, which ultimately leads to retarded liver regeneration in mice.^150^ Mechanistically, IL-4 produced by eosinophils binds directly to IL-4Rα on hepatocytes, promoting liver regeneration following PHx and toxin-induced damage. IL-4 administration is sufficient to drive quiescent hepatocytes into the cell cycle and enhance their proliferation, even in the absence of injury.^151^ IL-4-deficient mice are characterized by an almost complete lack of proliferation of hepatocytes after PHx.^148^ While IL-4Rα knockout (KO) in hepatocytes indeed reduces the direct response to IL-4, it does not completely inhibit liver regeneration. This observation suggests the involvement of alternative pathways that compensate for the lack of IL-4Rα. One plausible explanation is that IL-4 increases the ability of non-parenchymal cells, such as macrophages and hepatic stellate cells, to produce IL-6.^148,151^ This cytokine can then promote hepatocyte proliferation independently of IL-4Rα, highlighting the complex network of interactions that facilitate liver regeneration beyond single receptor-mediated pathways.

### Natural killer cells

Natural killer (NK) cells comprise nearly 30% of all intrahepatic lymphocytes in humans.^152^ The function of NK cells in liver regeneration is complex. NK cell infiltration into the remnant liver occurs after PHx.^153,154^ In early studies, NK cells were shown to inhibit liver regeneration by secreting IFN-γ.^98,141^ Further research revealed that increased co-inhibitory receptor T-cell Ig and ITIM domain (TIGIT) expression occurs on NK cells. TIGIT is involved in mediating NK cell self-tolerance to maintain regenerative hyperplasia by impeding NK cell activation and decreasing IFN-γ production.^155^ Interestingly, another study demonstrated that NK cells can promote liver regeneration by scavenging extracellular adenosine triphosphate (ATP) through the activation of specific P2 receptors, which explains why hepatocellular proliferation is markedly lower in immunodeficient mice lacking NK cells compared to wild-type mice after PHx.^156^ The controversial functions of NK cells in liver regeneration may be associated with subset heterogeneity and the activation status.^157^

### Neutrophils

Neutrophils are the most common type of leukocyte. Evidence has demonstrated the immune defense and proinflammatory mediator roles of these cells in various types of liver diseases.^158–160^ Recent research shows that hepatocytes release cholesterol and C-X-C motif chemokine ligand 1 (CXCL1), drawing neutrophils from the bone marrow to the injured liver, where they subsequently secrete HGF to enhance hepatocyte proliferation and support liver regeneration.^161^ Moreover, Brandel et al. reported that apoptotic extracellular vesicles released from residual liver tissue after PHx are cleared by circulating neutrophils, which increase the levels of progrowth factors such as HGF and FGF2 instead of proinflammatory cytokines to support regeneration in the liver remnant.^162^ Kwon et al. documented that treating mice with prednisolone inhibits macrophage- and neutrophil-mediated phagocytosis after CCl_4_-induced liver injury, which intensifies liver damage and hinders liver regeneration.^163^ This process may involve removing necrotic cell debris and the regression of inflammatory responses.

### Innate lymphoid cells

Innate lymphoid cells (ILCs), a recently discovered component of the innate immune system, are divided into three subsets: ILC1s, ILC2s, and ILC3s, distinguished by their unique surface markers, transcription factors, and effector cytokines.^98^ Elevated extracellular ATP levels stimulate ILC1s and NK cells to enhance IL-22 secretion through P2X1 receptor binding after PHx. Deletion of IL-22 impairs hepatocyte proliferation and exacerbates liver injury in mouse models.^164^ Similarly, plasma IL-22 levels are consistently elevated in patients undergoing major liver resection.^164^ Moreover, environment-induced eustress has been shown to increase ILC1 numbers via activation of sympathetic β-adrenergic signaling, ultimately promoting remnant liver regeneration.^165^

### Dendritic cells

The role of dendritic cells (DCs) in liver regeneration remains incompletely understood. Castellaneta et al. reported a marked increase in the number of CD11c^+^ DCs in the liver following PHx. Additionally, DCs upregulate IL-10 gene transcription while downregulating IFN-γ gene transcription.^166^ Given the inhibitory role of IFN-γ in liver regeneration, the relationship between DCs and the cell cycle warrants further investigation.

### Platelets

Early studies emphasized the role of platelets in hemostasis, but recent studies have shown that platelets also play a key role in liver tissue repair.^167^ Numerous studies have demonstrated that low platelet counts correlate with the occurrence of liver failure following PHx in humans.^168–170^ Pharmacal administration to increase platelet counts could contribute to liver regeneration.^171–174^ Evidence suggests that molecules present in platelets, such as HGF, serotonin, platelet-derived growth factor (PDGF), VEGF and insulin-like growth factor (IGF), are involved in directly mediating hepatocyte proliferation.^175,176^ Recent studies have revealed a correlation between the platelet count and coagulation during regeneration. Activated platelets can amplify the coagulation response and trigger thrombin-dependent intrahepatic fibrin(ogen) deposition. Fibrin(ogen) deposition further promotes the accumulation of platelets and drives liver regeneration after PHx.^177^

### Hepatocytes

After discussing the significant contributions of non-parenchymal cells to liver regeneration, it is crucial to focus on hepatocytes. These cells not only constitute the majority of liver tissue but also drive its regeneration through unique proliferative abilities. A key element in this regenerative capacity is the augmenter of liver regeneration (ALR), a protein secreted by hepatocytes. ALR was initially identified in extracts from regenerating liver tissue following PHx.^178^ Preclinical and clinical evidence supports several roles for ALR in liver regeneration. First, hepatic ALR contributes to lipid homeostasis by regulating lipolytic enzymes. Maintaining lipid balance is vital for providing energy and metabolic substrates during the regenerative process.^178–182^ Second, mitochondrial energy production, a prerequisite for cell proliferation, relies on ALR, which is crucial for maintaining mitochondrial intermembrane integrity and mitochondrial DNA biogenesis.^183,184^ Finally, activation of the ALR/G protein-coupled high-affinity receptor in rats increases TNF-α and IL-6 expression via the p38-MAPK pathway, thereby modulating the local immune microenvironment through macrophage activation and enhancing hepatic regeneration after PHx.^185^

## Cellular crosstalk in liver repair and regeneration

**Fig. 3:**
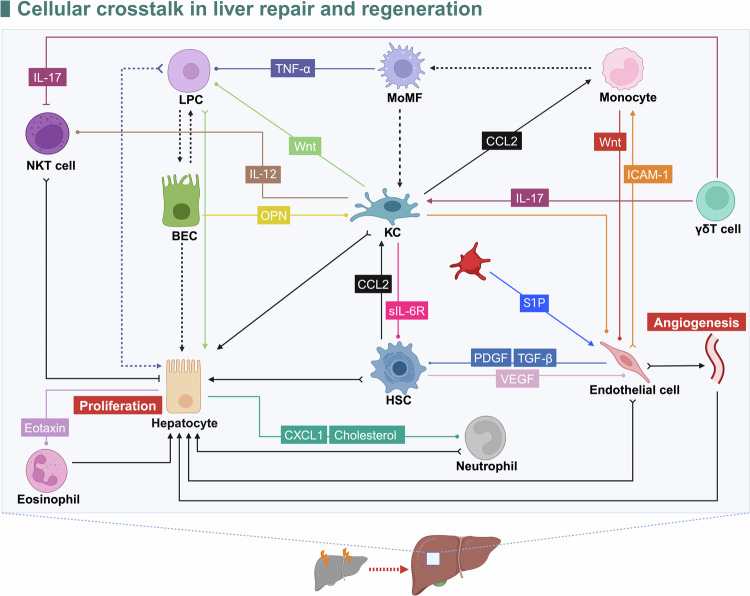
Crosstalk among diverse cells during liver repair and regeneration. Interactions between hepatocytes, immune cells, and other liver cell types, facilitated by the signaling molecules, regulate the dynamic process of liver regeneration. The dots represent relay cells. The tail of the Y-shaped arrow represents a continuous path with the previous relay. The arrows indicate a stimulatory effect on proliferation, and the T-arrows indicate an inhibitory effect. The dotted lines indicate dedifferentiation, differentiation, or transdifferentiation. HSC hepatic stellate cell, LPC liver progenitor cell, BEC biliary epithelial cell, NKT natural killer T, KC kupffer cell, IL interleukin, TNF-α tumor necrosis factor α; VEGF, vascular endothelial growth factor, PDGF platelet-derived growth factor, TGF-β transforming growth factor β, CCL2, C-C motif chemokine ligand 2, CXCL1 C-X-C motif chemokine ligand 1, S1P sphingosine 1-phosphate, sIL-6R, soluble IL-6R. ICAM-1, intercellular adhesion molecule 1, OPN osteopontin. The figure was generated with BioRender (https://biorender.com)

Recently, researchers have focused increasing attention on how the crosstalk of diverse cells affects liver regeneration. Accumulating evidence indicates the complexity and importance of cellular crosstalk in liver repair and regeneration (Fig. 3). The cellular response is crucial for signaling pathways to exert regulatory effects. By exploring cellular crosstalk and the underlying molecular mechanisms, we can gain a better understanding of how cell-to-cell communication affects the liver regeneration process.

### BECs and macrophages

Many studies have shown that BEC plasticity is essential for liver regeneration following extensive hepatocyte loss or impaired hepatocyte proliferation.^53,55,56,58–62^ Notably, crosstalk between BECs and macrophages also plays a key role in liver repair and regeneration. During injury recovery, macrophages engulf hepatocyte debris to maintain canonical Wnt signaling, which promotes the differentiation of BEC-derived LPCs into hepatocyte phenotypes rather than biliary phenotypes.^186^

Furthermore, BECs produce inflammatory mediators that recruit KCs to the injury area.^187–189^ In CCl_4_-induced liver injury, deletion of YAP/TAZ in BECs hinders the recruitment of phagocytic macrophages, impairing necrotic cell clearance and ultimately leading to regeneration defects at injury sites.^190^ Besides, BECs have been identified as the main source of osteopontin (OPN) following PHx, which activates macrophages to produce IL-6 and supports liver regeneration.^191–193^

### HSCs and endothelial cells

HSCs are located within the unique ecological niche of the liver, which allows HSCs to communicate with hepatocytes and multiple other non-parenchymal cells through direct contact and paracrine interactions. In particular, HSCs and endothelial cells exhibit close intracellular interactions that significantly influence liver regeneration. PDGF and TGF-β secreted by sprouting endothelial cells drive the accumulation and activation of HSCs around endothelial cells.^194–196^ Moreover, activated HSCs promote liver regeneration by regulating vessel remodeling and stabilization though paracrine proangiogenic signaling.^197^

### Macrophages and HSCs

KCs constitute the largest subset of immunocytes in the liver.^98^ When KCs sense liver injury, they activate a series of signaling pathways. Apart from directly stimulating hepatocyte proliferation, KCs also engage with other subsets of non-parenchymal cells, indirectly influencing and enhancing the liver regeneration process through cell‒cell interactions or paracrine signaling via the production of inflammatory factors.^86^

KCs play a crucial role in HSC mobilization and subsequent injury recovery.^125,198^ Inflammatory mediators, such as sIL-6R produced by activated KCs, induce HSCs to secrete HGF, which promotes hepatocyte proliferation.^88^ Notably, HSCs not only produce cytokines and growth factors that drive liver regeneration but also contribute significantly to maintaining liver architecture by regulating matrix remodeling.^197–200^

Reciprocally, HSCs regulate KC infiltration and activation during regeneration via CCL2.^86,201^ These recruited KCs, along with other immune cells, provide signals such as IL-13, which protect the liver through mechanisms dependent on HSC activation.^86^

### Macrophages and endothelial cells

Ordered angiogenesis is essential for liver regeneration, with impaired angiogenesis significantly delaying the overall process.^202^ Endothelial cells are critical for new vessel formation and paracrine signaling.^77,203,204^ KCs are strategically located in close proximity to endothelial cells within hepatic sinusoids, facilitating direct interactions between macrophages/monocytes and endothelial cells, which are crucial for stimulating endothelial growth during regeneration.^205,206^ In addition, resident KCs attract circulating monocytes to sites of injury via CCL2 and indirectly stimulate endothelial cells to upregulate adhesion molecules like intercellular adhesion molecule 1 (ICAM-1), thereby enhancing monocyte recruitment.^135,207,208^ This spatial relationship facilitates the recruitment of macrophages and activation of endothelial cells through various inflammatory mediators.^152,208–211^ Recent findings indicate that aging-mediated reprogramming of the crosstalk between endothelial cells and platelets promotes the infiltration of CXCR4^+^TIMP1^high^ macrophages, stimulates fibrosis, and impedes liver regeneration.^212^

Moreover, macrophages derived from infiltrating monocytes promote endothelial proliferation and vascular sprouting by secreting proangiogenic factors such as Wnt5a.^208^ In a model of PHx and acetaminophen (APAP)-induced acute liver injury (AILI), mice lacking CD11b on monocytes develop unstable vasculature, reduced regenerative capacity, and lower survival rates.^202,208^

### NKT/NK cells and macrophages

NKT and NK cells play crucial roles in mediating interactions between hepatic parenchymal and non-parenchymal cells.^137,155^ As an immunotolerant organ, the liver is continuously exposed to gut-derived bacterial products via the portal vein. Wu et al. reported that gut metabolites are involved in maintaining immune tolerance to regulate liver regeneration. In mice with intestinal dysbiosis, KCs induce NKT cell overactivation, leading to increased IFN-γ levels through enhanced IL-12 expression, ultimately inhibiting effective liver regeneration after PHx.^139^ Besides, deletion of myeloid phosphatase and tensin homolog (PTEN) results in increased M2 polarization of KCs and decreased NK cell activation. M2 KCs inhibit NK cell activation either through direct cell-to-cell contact or indirectly via cytokines, which subsequently promotes hepatocyte mitosis after PHx.^213^ In contrast, another study showed that cytokines secreted by NKT cells in the hepatic microenvironment may contribute to macrophage phenotype reprogramming, facilitating liver repair following IRI.^214^

### LPCs and macrophages

The regenerative process in chronic liver disease differs from that in acute liver injury. When hepatocyte proliferation is insufficient in individuals with chronic liver disease, alternatively activated LPCs can compensate by proliferating and differentiating into hepatocytes or cholangiocytes.^215^ The interaction between LPCs and macrophages is pivotal for regeneration. LPCs can recruit infiltrating macrophages to the site of injury.^135,215^ In a reciprocal manner, macrophages and other immune cells secrete TNF-α, IL-17, IL-22, IL-6, and TNF-like weak inducer of apoptosis (TWEAK), which stimulate LPC proliferation and differentiation into hepatocytes.^135,186,216–221^ Furthermore, this process of dynamic switch is tightly regulated by signaling pathways, particularly Notch and Wnt. Initially, Notch signaling mediates the dedifferentiation of BECs into LPCs, establishing a progenitor pool essential for initiating regeneration under severe hepatic stress.^53^ Subsequently, Wnt signaling directs LPC differentiation into functional hepatocytes, replacing damaged liver tissue and restoring organ function.^53,186,221^ Together, these pathways coordinate the cellular transformations necessary for an effective regenerative response.

### Adaptive immune system and macrophages

The effects of liver regeneration on the adaptive immune system, particularly cellular interactions, remain poorly understood. IL-17 produced by γδT cells is critical for recruiting and activating inflammatory cells.^132^ In response to IL-17, IL-6 secretion by KCs and DCs increases, while IFN-γ secretion by NKT cells decreases, indicating that γδT cells can influence other immune cells to regulate hepatic repair.^132,141,166^

Moreover, the spleen is rich in B cells.^222^ Cytokines and chemokines produced in the spleen can reach the liver via portal vein circulation.^223^ Behnke et al. reported that hepatocyte proliferation was reduced in splenectomized mice compared to controls. Further research indicated that B cells are essential for maintaining metallophilic CD169^+^ macrophages through Ltβ expression. Depletion of CD169^+^ cells impairs IL-6 production and hampers liver regeneration.^223^

### Platelets and endothelial cells

Platelets and endothelial cells play pivotal roles in liver regeneration, interacting closely to coordinate vascular remodeling and tissue recovery. Platelets can contact LSECs to induce LSEC proliferation and secrete VEGF and IL-6 via sphingosine 1-phosphate (S1P).^224,225^ A prospective clinical trial indicated that von Willebrand factor (VWF) released by activated endothelial cells has a direct positive effect on platelet accumulation and liver mass recovery following PHx.^226^ These interactions between platelets and endothelial cells creates a regenerative microenvironment that is essential for efficient liver repair and function restoration.

### Effects of hepatocytes on non-parenchymal cells

Following liver injury, hepatocytes release various chemokines that attract immune cells to the site of damage, playing a key role in regulating the balance between injury and repair.^135,227–230^ For instance, brahma-related gene 1 (BRG1) interacts with NF-κB to activate eotaxin transcription, which induces eosinophil infiltration and the subsequent secretion of IL-4, that drive hepatocyte proliferation.^150^ Moreover, CCL5, expressed by hepatocytes, can activate M1 macrophage polarization and impede liver repair following AILI.^119,120^ Additionally, hepatocytes release cholesterol and CXCL1 to recruit neutrophils, which secrete HGF to enhance hepatocyte proliferation.^161^ Beyond immune cell recruitment, hepatocyte-derived cholic acid triggers the release of Hedgehog ligands by HSCs, initiating Hedgehog signaling and promoting hepatocyte proliferation.^231^

## Canonical signaling pathways involved in liver repair and regeneration

**Fig. 4:**
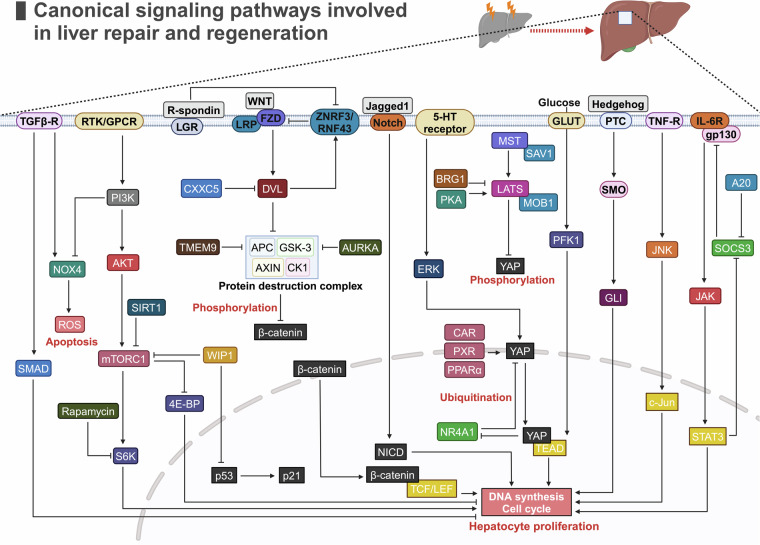
Canonical signaling pathways involved in liver repair and regeneration. This diagram illustrates the key molecular components and regulatory mechanisms involved in different signaling pathways. RTK receptor tyrosine kinase, GPCR G-protein-coupled receptor, LGR leucine-rich repeat-containing G protein-coupled receptor, LRP low-density lipoprotein receptor-related protein, FZD frizzled, 5-HT 5-hydroxytryptamine. GLUT glucose transporter type, SMO smoothened, PTC patched, IL-6R, IL-6 receptor, gp130 glycoprotein 130, TNF-R, TNF receptor, TGFβ-R TGF-β receptor, PI3K phosphoinositide 3-kinase, AKT protein kinase B, mTORC1 mammalian target of rapamycin complex 1, WIP wild-type p53-induced phosphatase 1, S6K ribosomal S6 protein kinase, 4E-BP eukaryotic translation initiation factor 4E-binding protein, CXXC5 CXXC5-type zinc finger protein 5, DVL disheveled, ZNRF3, Zinc and Ring Finger 3, RNF43 Ring Finger Protein 43, TMEM9 transmembrane protein 9, APC adenomatous polyposis coli, GSK-3 glycogen synthase kinase 3, AXIN axis inhibition protein, CK1 casein kinase 1, AURKA aurora kinase A, MST ste20-like kinase, SAV salvador, LATS large tumor suppressor kinase, YAP Yes-associated protein, AMPK AMP-activated protein kinase, BRG1 brahma-related gene 1, CAR androstane receptor, PXR pregnane X receptor, PPARα peroxisome proliferator-activated receptor α, PFK1 phosphofructokinase-1, GLI zinc finger protein, SMAD suppressor of mothers against decapentaplegic, NOX4 NADPH oxidase 4, ROS reactive oxygen species, NICD Notch intracellular domain, TCF T-cell factor, LEF lymphoid enhancer factor, NR4A1 nuclear receptor subfamily 4 group A member 1, TEAD TEA domain family member, JAK Janus kinase, JNK c-Jun N-terminal kinase, SOCS3 suppressor of cytokine signaling 3, STAT3 signal transducer and activator of transcription 3. (The figure was generated with BioRender (https://biorender.com)

Signaling pathways play crucial roles in liver repair and regeneration, orchestrating key processes such as differentiation, proliferation, metabolism and cell death to maintain hepatic health and function. These pathways are not only central to normal physiological liver functions but also have significant impacts on liver disease progression. Therefore, a deep understanding of these signaling pathways is highly important for both basic research and the development of new therapeutic targets for liver diseases (Fig. 4).

### Wnt/β-catenin signaling pathway

The Wnt signaling pathway plays an essential role in physiological homeostasis and pathobiological repair in the liver.^232–234^ Wnt ligands, which are glycoproteins, are mainly produced by KCs and endothelial cells and secreted with the assistance of the cargo protein Wntless (WLS).^235,236^. Wnt signaling is initiated when Wnt ligands bind to Frizzled (FZD) receptors. The diversity of Wnt ligands and FZD receptors results in specificity and complexity in Wnt pathway activation.^237,238^ The most studied pathway in liver repair and regeneration is canonical Wnt signaling, which is mediated by β-catenin.^239^ Interestingly, β-catenin can also be activated by signals besides Wnt, including HGF, EGF, and protein kinase A (PKA). However, the dual deletion of the coreceptors LRP5/6 delays liver regeneration, underscoring the critical role of Wnt ligands in activating β-catenin during this process.^113^

In the canonical Wnt/β-catenin pathway, the protein destruction complex (adenomatous polyposis coli [APC], casein kinase 1 [CK1], glycogen synthase kinase 3 [GSK-3], and axis inhibition proteins [AXINs]) targets and phosphorylates the β-catenin protein. Phosphorylated β-catenin is subsequently recognized by β-transducin repeat-containing protein (βTRCP), leading to its degradation.^238^ However, Wnt binding FZD receptors and the redundant coreceptors low-density lipoprotein receptor-related proteins (LRP)5/6 together activate dishevelled (DVL). Then, DVL restrains the protein destruction complex; the β-catenin protein is protected from degradation and subsequently translocated into the nucleus. β-Catenin ultimately activates downstream target genes via T-cell factor/lymphoid enhancer-binding factor (TCF/LEF).^238^

#### Function

The activation of canonical Wnt signaling is critical for coordinating liver repair and regeneration after liver resection or toxicant injury.^238–240^ Unlike in murine hepatocytes, the Wnt/β-catenin pathway plays an indispensable role in the mitosis of human hepatocytes.^241^ β-Catenin expression is upregulated within minutes after PHx and persists for up to 24 hours, which then results in increased β-catenin nuclear translocation. The β-catenin-TCF4 complex subsequently activates target genes such as *Ccnd1* and initiates the G1-to-S phase transition.^242^ Disturbances of the Wnt-β-catenin pathway, including β-catenin KO, WLS KO, and LRP5/6 KO, result in decreased Cyclin D1 expression and impaired liver regeneration.^243,244^

Furthermore, Wnt/β-catenin signaling is a major regulator of hepatic metabolic zonation. The heterogeneity of zones allows cells to perform specific functions effectively in response to various injuries.^245,246^ Disruption of the Wnt/β-catenin pathway interferes with metabolic zonation.^247,248^

#### Regulatory mechanisms

Production of Wnt ligands is crucial for activating and regulating the Wnt/β-catenin signaling pathway. As previously shown, endothelial cell-derived Wnt ligands play crucial roles in the regulation of liver zonation and regeneration.^249^ Spatial omics further revealed that cell membrane protein tyrosine kinase with immunoglobulin-like and EGF-like domains (TIE)-1 is necessary for endothelial cells to produce Wnt and assist in liver regeneration following PHx.^250^

Any alteration in the activity of the protein destruction complex invariably impacts the Wnt/β-catenin signaling pathway. Transmembrane protein 9 (TMEM9), which is localized mainly in lysosomes, is highly expressed in the pericentral zone during the liver regenerative phase. TMEM9 downregulates APC expression and disrupts the protein destruction complex though v-ATPase-induced lysosomal protein degradation, which prevents β-catenin degradation.^251^ However, aurora kinase A (AURKA), an important mitosis regulator, increases the phosphorylation of GSK-3β and inactivates the protein destruction complex, which stimulates the upregulation of β-catenin expression and promotes liver regeneration.^252,253^ Consistent with these findings, an APAP overdose model revealed that increased GSK-3β phosphorylation contributes to decreased β-catenin ubiquitination and proteolysis.^234,254^

Influencing the activity of DVL also impacts the signaling pathway. The cell-surface transmembrane E3 ubiquitin ligases Zinc and Ring Finger 3 (ZNRF3) and Ring Finger Protein 43 (RNF43) act as negative feedback regulators of Wnt/β-catenin signaling by inducing the degradation of FZD and LRP6 in a DVL-dependent manner, and R-spondin (RSPO) ligand binding to leucine-rich repeat-containing G protein-coupled receptor (LGR) 4/5 receptors can clear ZNRF3 and RNF43 to strengthen Wnt/β-catenin signaling.^255–257^ Liver-specific deletion of *Lgr4* significantly restrains liver regeneration. Moreover, additional RSPO supplementation in LGR4 KO mice fails to rescue hepatocyte proliferation.^246^ This finding is consistent with the observation that double KO of ZNRF3*/*RNF43 promotes liver regeneration. Further RNA-Seq analysis demonstrated that the expression of target genes related to Wnt signaling are downregulated in LGR4 KO mice.^246^ These findings underscore the importance of the RSPO–LGR4–ZNRF3/RNF43 axis in the Wnt/β-catenin-mediated regenerative response.^246,258^ However, excessive activation of Wnt/β-catenin signaling is a feature of liver tumors.^259–261^ Zone 3, also termed the pericentral zone, is highly susceptible to hepatocarcinogenesis, partly due to the activation of Wnt/β-catenin signaling.^259,262,263^ Hepatocyte-specific deletion of *Znrf3/Rnf43* increases the predisposition to hepatocellular carcinoma (HCC) due to an imbalance between differentiation and proliferation.^264^ Importantly, the harmonious cooperation of ZNRF3 and RNF43 restricts Wnt/β-catenin activity and maintains homeostasis between proliferation and tumor formation.^265^ Likewise, CXXC5-type zinc finger protein 5 (CXXC5) negatively regulates the Wnt/β‐catenin pathway by directly interacting with DVL.^266,267^ Disruption of the CXXC5/DVL interaction ameliorates metabolic dysfunction and enhances the regenerative capacity in a Wnt/β-catenin-dependent manner.^266^

Additionally, it is equally crucial to regulate senescence-inducing signals downstream of the Wnt/β-catenin cascade. A 3D spheroid model of primary human hepatocytes revealed that Wnt/β‐catenin signaling induces major hepatocyte proliferation by suppressing the p53‐PAI1 signaling axis.^268^

Moreover, several factors that influence receptor distribution also impact downstream Wnt signaling effects. serine palmitoyltransferase (SPT), which is involved in sphingolipid biosynthesis, affects the cellular distribution of β-catenin by regulating cadherin-mediated adherens junctions.^269,270^ These findings provide a potential target for promoting liver regeneration.

While disruptions in the Wnt/β-catenin signaling pathway can significantly impact liver regeneration, the liver possesses compensatory mechanisms to overcome these challenges. Hepatocyte-specific β-catenin KO inhibited the expression of *Ccnd1* in the early stage after PHx.^243,271^ However, this defect in liver regeneration was compensated by the activation of mTORC1 at 72 h post-hepatectomy. Further analysis revealed that insulin is the upstream driver that activates mTORC1 in β-catenin-deficient mice.^272^

### Hippo/YAP signaling pathway

The Hippo signaling pathway, a highly conserved signaling pathway in mammals, contributes to regulating cell proliferation, survival, and differentiation to control the organ size, tissue regeneration and tumorigenesis.^273^ The Hippo pathway and its components were first identified in *Drosophila melanogaster*.^274^ The core of the pathway is Ste20-like kinase 1/2 (MST1/2). Upon activation, the Hippo pathway triggers the phosphorylation of the MST1/2-salvador1 (SAV1) complex via upstream activators, including neurofibromatosis 2 (NF2), RhoA and Kibra.^275^ MST1/2 then phosphorylates and activates large tumor suppressor kinase 1/2 (LATS1/2) with the assistance of the adaptor protein MOB1a/b.^276,277^ The activation of core components of the Hippo pathway leads to the phosphorylation of specific serine residues in YAP/TAZ. Phosphorylated YAP/TAZ are subsequently degraded and sequestered in the cytoplasm and fails to translocate from the cytoplasm to the nucleus. However, the absence of Hippo signaling triggers unphosphorylated YAP/TAZ to translocate into the nucleus and activate mainly the transcriptional enhanced associate domain (TEAD) family of transcription factors to drive Hippo target gene expression in the liver.^277–279^ Downstream target genes related to liver repair and regeneration can be directly activated by the YAP/TAZ signaling pathway.^280^

#### Function

In the early stage following PHx, a reduction in the phosphorylation of YAP and an increase in the nuclear localization of YAP are observed in hepatocytes.^281,282^ Liver-specific KO of upstream Hippo kinases, including MST1/2, LATS1/2, and the adaptor proteins SAV1 and MOB1a/b, leads to decreased YAP phosphorylation and increased nuclear translocation. Sustained YAP activation eventually triggers hepatocyte hyperproliferation and liver enlargement.^280,283–285^

Liver deficiency of YAP, or both YAP and TAZ, results in abnormalities in bile duct morphogenesis, hepatitis and fibrosis due to cholestatic injury.^286^ The deletion of YAP/TAZ in adult BECs, but not in hepatocytes, causes bile acid overload and impairs liver regeneration in response to CCl_4_-induced liver injury.^190^ Notably, BECs can promote the reconstitution of the biliary system to participate in liver repair through DR.^57,287^ Paz et al. reported that YAP signaling is essential for DR following injury, with mTORC1 potentially acting as an intermediate link in YAP-mediated liver repair.^288,289^

#### Regulatory mechanisms

Accumulating evidence suggests bidirectional regulation between YAP and metabolism, especially glucose metabolism. YAP can drive glucose uptake and glycolysis partially via the glucose transporter (GLUT).^290^ Subsequently, the increased uptake of glucose promotes the binding of YAP/TAZ to TEAD transcription factors in collaboration with the key glycolysis gene phosphofructokinase-1 (PFK1), consequently increasing cell proliferation.^291,292^

Crosstalk also exists between the YAP pathway and gluconeogenesis. Glucagon induces the production of cyclic adenosine monophosphate (cAMP) and thereby activates PKA by binding to a G-protein coupled receptor (GPCR), which ultimately activates gluconeogenesis. Interestingly, PKA-induced by glucagon also activates LATS1/2 and suppresses YAP.^293,294^ YAP can inhibit glucose-6-phosphatase catalytic subunit (G6PC) and phosphoenolpyruvate carboxykinase 1 (PCK1) via peroxisome proliferator-activated receptor gamma coactivator 1 (PGC-1α), eventually hampering gluconeogenesis.^295^

The YAP pathway and lipid metabolism pathways reciprocally modulate the activity of the other. The deletion of *Lats2* or *Mst1* may trigger the accumulation of cholesterol by increasing sterol regulatory element binding protein (SREBP) activity.^296,297^ Moreover, steatosis triggered by the absence of PTEN can be compensated by the overexpression of MST1.^298^ Mevalonic acid, a precursor of cholesterol, can be catalyzed by HMG-CoA reductase. However, additional products of the mevalonate pathway, such as geranylgeranyl, likely contribute to inhibiting the Hippo signaling pathway and increasing YAP translocation via posttranslational modifications.^278,299^

YAP/TAZ is involved in a variety of processes that mediate liver regeneration. Certain nuclear receptor agonists, including constitutive androstane receptor (CAR), pregnane X receptor (PXR) and peroxisome proliferator–activated receptor α (PPAR), contribute to hepatocyte growth and proliferation by promoting the nuclear translocation of YAP.^300–302^ Additionally, YAP contributes to 5-HT-mediated liver regeneration partly via pERK activation.^303^ Liver-specific BRG1 KO can also positively induce LATS1 expression, activate Hippo signaling, and inhibit cell cycle progression.^304^

Interestingly, the Hippo pathway regulates cell proliferation through mechanosensing within tissues, including changes in the physical state between cells and the cell matrix.^305,306^ It is responsible for mediating the inhibition of cell growth induced by direct cell–cell contact.^305^ A recent study revealed that the cell adhesion molecule KIRREL1 contributes to the recruitment of SAV1 to cell‒cell contact sites and facilitates the activation of downstream kinases in the Hippo pathway. This process is regulated by YAP-induced KIRREL1 expression, forming a feedback loop.^307^ Moreover, beyond the increased shear forces sensed by endothelial cells and HSCs to secrete more HGF after PHx, these extracellular stimuli can be sensed directly by hepatocytes and result in the activation or inactivation of YAP.^71,81,92,308^ Specifically, β1 integrin, a shear force sensor on the cell membrane, activates its downstream effector FAK to reduce the phosphorylation of LATS, which drives hepatocytes to enter the cell cycle via a YAP-dependent mechanism.^308^

Mechanistically, several posttranslational modifications of proteins, including phosphorylation, ubiquitination, acetylation, O-GlcNAcylation, and methylation, are involved in the regulation of key Hippo/YAP signaling processes by integrating factors and other signaling pathways. Among them, phosphorylation is the most widely studied and is regulated by the STRIPAK-PP2A complex, which dephosphorylates Hippo/MST kinases; PP1, which dephosphorylates TAZ and LATS1; TNF-α, which phosphorylates YAP; the CGRP receptor component RAMP1, which decreases YAP phosphorylation; and PPM1A/PP2Cα, which directly dephosphorylates YAP and promotes the nuclear distribution of YAP.^309–313^ Moreover, nuclear receptor subfamily 4 group A member 1 (NR4A1) promotes the ubiquitination and degradation of YAP via negative feedback to maintain liver homeostasis.^314^ K48-linked YAP ubiquitination induced by p300 results in proteasomal degradation in the cytoplasm. However, liver enlargement and regeneration activated by PXR cause YAP to undergo deacetylation and K63-linked ubiquitination in a Sirt2-dependent manner, which allows YAP-TEAD binding in the nucleus.^315^ Moreover, O-GlcNAcylation of YAP by O-GlcNAc transferase disrupts LATS1/2-induced YAP phosphorylation.^316^ And an extracellular high-glucose environment can drive YAP activity by modifying YAP with O-linked β-N-acetylglucosamine.^317^ Importantly, the methylation of YAP at K494, which is mediated by the SET1A methyltransferase complex, controls the activation of YAP by preventing its export from the nucleus to the cytoplasm.^318^

### Notch signaling pathway

The Notch pathway is a conserved signaling pathway that plays roles in cell fate decisions, homeostasis maintenance, and liver regeneration.^319^ Four types of Notch signaling receptors have been identified in mammals. The liver is rich in the Notch1 and Notch2 receptors, which are expressed mainly on BECs and LPCs.^320^ Ligand Jagged1 can activate Notch receptors.^321^ Ligand–receptor binding results in the release of the Notch intracellular domain (NICD) and NICD interaction with recombination signal-binding protein immunoglobulin kappa J (RBPJ) to initiate the transcription of downstream target genes such as HES/HEY.^322^

#### Function

The Notch signaling pathway plays a pivotal role in the formation and remodeling of the biliary tree.^323,324^ Defective hepatic Notch signaling in both humans and mice leads to bile duct abnormalities and cholestasis.^325–327^ The expression of Notch receptors is also upregulated during injury repair. Following large-scale liver injury, Notch signaling upregulates the expression of the insulin-like growth factor 1 receptor (IGF1R) in BECs and sensitizes BECs to IGF1, which promotes IGF1-induced BEC proliferation.^328^ Moreover, the activation of Notch signaling promotes the differentiation of LPCs into BECs, whereas the inactivation of Notch signaling shifts LPCs toward hepatocytes.^323,328,329^ Interestingly, a dual genetic lineage tracing approach revealed the mechanism underlying the origin of LPCs. The inhibition of Notch signaling induces the dedifferentiation of BECs to LPCs, whereas the activation of Notch promotes BEC proliferation. This BEC-to-LPC conversion contributes to tissue repair when hepatocytes are senescent or after severe liver injury.^53^

Some studies have highlighted the direct role of Notch signaling in hepatocyte proliferation. The translocation of the NICD to the nucleus increases in the early stage after PHx.^330^ Silencing of Notch1 and Jag1 disturbs cell cycle progression to delay liver regeneration.^330,331^ This may involve the NICD/protein kinase B (AKT)/HIF-1α pathway.^331^

#### Regulatory mechanisms

Forkhead box protein O1 (FOXO1) has been found to activate Notch signaling and regulate cellular differentiation in myoblasts by facilitating Notch binding to the *Hes1* promoter and activating downstream target genes.^332^ Furthermore, FOXO1 and Notch coordinately regulate gluconeogenesis by modulating G6PC expression. Sustained activation of FOXO1 results in increased hepatic glucose production and insulin resistance.^333^ Interestingly, the suppression of Notch signaling can improve insulin resistance in a FOXO1-dependent manner, suggesting a potential therapeutic avenue.^334^

Many studies have explored the crosstalk between Hippo signaling and the Notch signaling pathway in tumor research models.^335,336^ It has been reported that the downstream molecule Notch1 is a downstream target of YAP/TAZ in liver regeneration. This activation of the YAP/TAZ-Notch1-NICD axis contributes to liver regeneration following PHx.^337^ Lu et al. reported that the Notch and Hippo signaling pathways coordinate to regulate the differentiation of LPCs.^320^ Apart from competitively binding to the promoter region of YAP, RBPJ triggers the downregulation of YAP expression and inhibits YAP nuclear translocation in LPCs. Activation of the Notch–RBPJ–YAP axis contributes to promoting LPCs differentiation into BECs.^320^

### Hedgehog signaling pathway

#### Function

Hedgehog is known to contribute to repair and regenerative processes in a paracrine manner after injury. Mechanistically, Hedgehog binding to the transmembrane protein patched (PTC) induces the activation of smoothened (SMO), which stimulates the expression of downstream genes such as *Ccnd1* via zinc finger transcription (GLI) factors.^338–340^ Several studies have shown that aging inhibits liver regeneration following PHx.^212,341^ Consistent with this, transcriptomic analysis revealed that the Hedgehog signaling pathway is the most differentially activated pathway in young versus old hepatocytes after PHx,^342^ which has also been verified in the context of diabetes-mediated liver disease.^343^

#### Regulatory mechanisms

In models of regeneration following various types of liver injury, increased Hedgehog expression has been observed. Nuclear phosphorylated c-Jun N-terminal kinase (JNK) in HSCs triggers Hedgehog production, which promotes regeneration via GLI1-mediated Cyclin D1 expression after ALPPS.^344^ Similarly, miR-182-5p overexpression in hepatocytes increases cholic acid production by activating cholesterol 7 alpha-hydroxylase (CYP7A1), driving HSCs to secrete Hedgehog and stimulating hepatocyte proliferation following PHx.^231^ Additionally, Hedgehog signaling regulates DR cell fate and injury repair through the GLI/YAP pathway in response to CCl_4_-induced liver injury.^345^

### TGF-β signaling pathway

#### Function

TGF-β signaling ensures that liver regeneration occurs in a controlled manner in multiple ways. TGF-β expression increases at 4 h and peaks at 72 h after PHx.^346^ ROS immediately trigger endothelial cells to produce the matricellular protein thrombospondin-1 (TSP-1), which contributes to the conversion of TGF-β1 into its active form following PHx.^347^ Activated TGF-β binds to its transmembrane receptor (TβR), subsequently inducing the phosphorylation of suppressor of mothers against decapentaplegic (SMAD) and its translocation into the nucleus, which suppresses hepatocyte proliferation by inhibiting the expression of downstream DNA synthesis-related genes.^348^

TGF-β signaling is also involved in regulating cell fate conversion during liver repair. Severe liver injury exceeding a threshold for hepatocyte replication leads to the initiation of the repair response. Specifically, although the excessive accumulation of myofibroblasts (MFs) leads to defective repair and liver fibrosis, transient HSC-derived MFs are important for replacing the large amount of parenchymal cells that were lost, especially in the context of chronic liver injury.^349^ TGF-β stimulation allows HSCs to exhibit LPC features via the Jagged1/Notch pathway.^350,351^ Additionally, TGF-β signaling drives compensatory hepatocyte-mediated cholangiocyte transdifferentiation to reconstruct the intrahepatic biliary system in mice with NOTCH signaling defects.^352^

However, during chronic liver injury, this compensatory may lead to abnormal biliary structures and disrupted bile acid transport through the TGF-β receptor 1 (TGFβ-R1)–β-catenin signaling pathway.^353^

#### Regulatory mechanisms

TGF-β signaling is involved in promoting apoptosis by regulating oxidative stress.^354^ A decrease in NADPH oxidase 4 (NOX4) levels during liver regeneration has recently been recognized as a significant source of ROS.^355^ TGF-β-induced hepatocyte apoptosis is dependent on NOX4.^356^

Transcriptome analysis revealed that NOX4 KO mice exhibit accelerated liver mass recovery and increased hepatocyte proliferation following PHx. This is associated with increased expression of MYC and decreased expression of the TGF-β.^357^ Further studies revealed TGF-β/NOX4-mediated apoptotic effect can be repressed by the mitogens EGF and HGF. Specifically, EGF blocks the upregulation of NOX4 expression in a phosphoinositide 3-kinase (PI3K)-dependent manner.^358^ However, the transient escape of early regenerating hepatocytes results in a reduced response to TGF-β inhibition.^359^ The spatiotemporal regulation of TGF-β signaling has not yet been clarified. Potential resistance mechanism may involve intracellular glutathione-mediated antioxidants, which contribute to blocking ROS production induced by TGF-β.^235,360,361^ Another hypothesis is that hepatocytes undergo a transient epithelial–mesenchymal transition to escape the suppressive effects of elevated TGF-β levels. TGF-β is widely recognized for its potent ability to induce the epithelial–mesenchymal transition in various cell types, including hepatocytes.^362^

Notably, signaling crosstalk also plays a role in this regulation. The positive interaction between TGF-β signaling and the Hippo/YAP pathway is highlighted by increased YAP1 translocation after PHx, which promotes the physical accumulation of pSMAD2.^363,364^

### PI3K/AKT signaling pathway

#### Function

The PI3K family, which includes numerous kinases involved in signal transduction via receptor tyrosine kinases (RTKs) and GPCRs, plays a role in liver regeneration in response to ligands, including the growth factors HGF, EGF, and TGF-α and the cytokines TNF-α and IL-6.^365^ When the RTK/GPCR is activated, PI3K catalyzes the production of phosphatidylinositol-3,4,5-triphosphate, subsequently recruiting and phosphorylating specific signaling proteins such as AKT.^366^ PI3K subunit KO in mice or treatment with a PI3K inhibitor markedly aggravated hepatocyte necrosis and inhibited liver regeneration.^365,367^ mTORC1 is an effector of the PI3K-AKT pathway during regeneration.^368,369^ mTORC1 is involved in the regulation of both cell growth and proliferation via the activation of ribosomal S6 protein kinase 1 (S6K). The inhibitor rapamycin can disrupt the proliferation process by suppressing S6 kinase activation.^370^ Additionally, mTOR1 also phosphorylates and inhibits eukaryotic translation initiation factor 4E-binding protein 1 (4E-BP1), which downregulates the expression of Cyclin D1.^371^

Among the RTKs involved in liver regeneration, mesenchymal-epithelial transition factor (c-MET) and epidermal growth factor receptor (EGFR) are the most well-known. HGF has been shown to be closely associated with liver repair in response to liver injury. Various non-parenchymal cells, such as LSECs, KCs, HSCs and neutrophils, secrete HGF.^372^. HGF, as the exclusive ligand for c-MET, can phosphorylate c-MET and recruit signaling molecules. Mechanistically, the HGF/C-Met signaling pathway induces the activation of multiple downstream signaling cascades, including the JAK/STAT3, Ras/Raf, and PI3K/AKT/NF-κB pathways, throughout the liver repair process.^373^

The EGFR signaling pathway is another key pathway that regulates liver regeneration. The ligands of EGFR that are involved in liver regeneration include EGF, heparin-binding EGF-like growth factor (HB-EGF), amphiregulin and TGF-α.^372^ The redundancy of multiple ligands ensures the activity of the EGFR signaling pathway. Notably, a compensatory mechanism exists between EGFR and MET signaling. Individual KO delays but does not completely inhibit liver regeneration. However, combined KO affects the activation of mTOR and AKT and inhibits regeneration.^374^

#### Regulatory mechanisms

The regulation of mTORC1 activity is critical for influencing the PI3K/AKT pathway.

Alongside its regulation of glucose homeostasis and lipid metabolism, the histone deacetylase sirtuin (SIRT1) has been reported to act as a negative regulator of mTORC1.^375^ Additional SIRT1 administration can even rescue the regenerative capacity of aged mice.^376^ The oncogene wild-type p53-induced phosphatase 1 (WIP1) has been found to play dual roles in liver regeneration. WIP1 can not only dephosphorylate and inactivate mTOR but also inhibit the p53/p21 pathway following PHx. However, the pro-regenerative role of the mTORC1/S6K signaling pathway overwhelms the antiproliferative role of the p53/p21 signaling pathway in *Wip1* deficient mice.^377^ Moreover, negative regulation was likewise identified. Apoptosis-stimulating protein two of p53 (ASPP2), the binding partner of p53, has been found to suppress liver regeneration by inhibiting the mTORC1 pathway.^378^

Crosstalk between multiple signaling pathways also contributes to the regulation of liver regeneration. While IL-6/STAT3 is crucial, compensatory hepatocyte hypertrophy via the PI3K/AKT pathway has been observed in conditional STAT3 KO mice.^379^

The AKT/mTORC1 axis influences the functional transformation of non-parenchymal cells. The phenotypic transformation of LSECs regulates the balance between regeneration and fibrosis. This conversion is dependent on the ERK/AKT axis.^380^ Activated ERK1/2 switches LSECs to a pro-regenerative phenotype by releasing HGF and Wnt, whereas activated AKT triggers the profibrotic phenotype of LSECs. Further work revealed that AKT can reduce the activity of ERK1/2 via mTOR.^380^ These findings provide a potential target for improving liver regeneration in patients with liver fibrosis.

Recently, Lao et al. reported the AKT/mTORC1 axis influences the functional transformation of non-parenchymal cells, including LSECs. The ERK/AKT axis regulates LSEC phenotypes, balancing regeneration and fibrosis.^380^ Activated ERK1/2 promotes a pro-regenerative phenotype by releasing HGF and Wnt, while activated AKT induces a profibrotic phenotype. AKT can also reduce ERK1/2 activity via mTOR, offering a potential target for improving liver regeneration in patients with fibrosis.^380,381^

### Signaling pathways associated with inflammatory cytokines

#### TNF-α signaling pathway

The TNF-α signaling pathway, a critical part of the initiation phase, promotes liver repair and regeneration through the synergy of different liver cells. Inactivated KCs maintain NF-κB in the cytoplasm via the inhibitory KB protein (IKB), whereas in activated KCs, the inhibitory KB kinase (IKK) phosphorylates IKB, releasing NF-KB into the nucleus in response to various extracellular stimuli, such as LPS and complement C3/C5. Subsequently, KCs initiate the production of TNF-a and IL-6, and secreted TNF-a can bind to TNF receptor 1 (TNF-R1) and amplify this reaction in an autocrine manner.^97,98^

Beyond its indirect role in regulating liver regeneration in KCs, TNF-α also directly stimulates hepatocytes to activate JNK. JNK then phosphorylates the transcription factor c-Jun, initiating the expression of genes related to the cell cycle.^382,383^

#### IL-6 signaling pathway

The IL-6/STAT3 signaling pathway is critical for cellular responses in the early stage of liver regeneration. IL-6 is responsible for activating 40% of the genes that are not expressed in normal livers but are expressed after hepatectomy.^383,384^ Once IL-6 binds to IL-6R, the IL-6/IL-6R complex is able to activate its coreceptor gp130, which initiates downstream pathways, including the JAK/STAT3, PI3K/AKT, and MAPK pathways.^2^

Activated JAK induces the phosphorylation of STAT3, which translocates to the nucleus, where it promotes the expression of cell proliferation-related genes. STAT3 can negatively regulate gp130 expression by inducing suppressor of cytokine signaling (SOCS).^2^ The A20 protein is involved in the regulation of this feedback inhibition. In conjunction with inhibiting NF-κB activation to block inflammation, A20 can enhance the IL-6/STAT3 signaling pathway and trigger hepatocyte proliferation by decreasing SOCS3 expression.^385^

In the dynamic process of liver repair and regeneration, cellular crosstalk plays a pivotal role in orchestrating the complex interactions among various cell types within the hepatic microenvironment. Efficient recovery of liver function post-injury relies heavily on the activation and regulation of several key signaling pathways. These pathways govern the responses of individual cell types and facilitate critical intercellular communication, which is essential for regeneration. Further investigation into these signaling pathways and their roles in mediating interactions between parenchymal and non-parenchymal cells is necessary to deepen our understanding of liver regeneration mechanisms.

## Reprogramming of metabolic pathways in liver repair and regeneration

The liver plays a central role in regulating the homeostasis of multiple metabolic pathways, especially glucose, lipid, and amino acid metabolism pathways. Metabolites serve as precursors, intermediates, and final products of cellular processes.^386^ Metabolic activities are orchestrated by a delicate interplay among cellular signaling pathways. Recent studies have shown that cell-intrinsic metabolic remodeling is necessary to support cellular phenotypic changes and effector functions in response to various injuries. The metabolic response influences the signaling molecules and pathways involved in the regulation of liver repair and regeneration. Reprogrammed cellular metabolic networks contribute to meeting the energy demand, providing anabolic precursors and generating molecular signals that initiate regenerative processes.^387,388^ Here, we discuss critical targets and potential strategies aimed at the metabolic reprogramming of liver cells that may regulate the progression of liver repair and regeneration (Table 1).

**Table 1:** Metabolic network alterations during liver repair and regeneration

Metabolic type	Target	Location	Metabolic signaling pathways	Alterations in metabolic networks	Effect	Ref.
Gluconeogenesis	AKT	Hepatocyte	AKT/FOXO1/G6PC + PCK1	insulin sensitivity ↓, glycogenolysis ↓, gluconeogenesis ↓	liver regeneration ↓	^295,399^^,^^402,404^
	AnxA6	Hepatocyte	AnxA6/PEPCK	alanine uptake ↑, gluconeogenesis ↑	liver regeneration ↑	^408^
	AQP9	Hepatocyte	AQP9/GK/GPD1, AQP9/GS	glycerol uptake ↑, gluconeogenesis ↑, glycogen synthase ↑ , ATP ↑, oxidative stress ↓	liver regeneration ↑	^409^
Glycogen synthesis	GSK-3	Hepatocyte	AKT/GSK-3/GS	glycogen synthesis ↓, insulin sensitivity ↓	liver regeneration ↓	^391,415^
	CDK5RAP3	Hepatocyte	CDK5RAP3/CPT1α + FASN	glycogen synthesis ↑, oxidation of fatty acids ↑, fatty acids synthesis ↑	liver regeneration ↑	^417^
	HNF4α	Hepatocyte	HNF4α/Cyclin D1	glycogen synthesis ↓ , glucose uptake ↓	liver regeneration ↓	^419–421^
Glycolysis	PP2A	Hepatocyte	PP2A/AKT/GSK3β, PP2A/PFKFB2	glycolysis ↑	liver regeneration ↓	^424,425^
	FXR	Hepatocyte	-	glycolysis ↑, OXPHOS ↓	liver regeneration ↓	^427^
	PDK4	Hepatocyte	PDK4/ Insulin/AKT, PDK4/GCK, PDK4/AMPK/FOXO1/CD36	glycolysis ↓, insulin sensitivity ↓, fatty acids uptake ↓ , oxidation of fatty acids ↓, ATP ↓	liver regeneration ↓	^428^
	PKM2	Macrophage	PKM2/HIF-1α	glycolysis ↑, OXPHOS ↓	liver regeneration ↓	^434,435^
	FOXO1	Macrophage	-	glycolysis ↑, OXPHOS ↓ , ATP ↓	liver regeneration ↓	^442^
Fatty acids synthesis	NCOR1	Hepatocyte	NCOR1/FASN + ACC, NCOR1/GLUT4 + G6PD	fatty acids synthesis ↓, glucose uptake ↓, PPP ↓	liver regeneration ↓	^451^
	ACC	Hepatocyte	EGFR/ AMPK/ACC	fatty acids synthesis ↑	liver regeneration ↑	^374^^,452,453^
	RNF43/ZNRF3	Hepatocyte	RNF43 + ZNRF3/Wnt	fatty acids synthesis ↓, disrupted lipid zonation	Defective termination of liver regeneration	^264,265^
Fatty acids oxidation	NRF2	Hepatocyte	HGF + EGF/NRF2/PARK/PTEN/PPARα + CPT1α	ROS↓ oxidation of fatty acids ↑, ATP ↑	liver regeneration ↑	^43,418^^,459,461^
SCFAs	SCD1	Hepatocyte	FASN + SCD1/phospholipid synthesis	fatty acids synthesis ↑	liver regeneration ↑	^470^
Eicosanoids	COX2	KC, Hepatocyte	COX2/PG	PG ↑	liver regeneration ↑	^474–476^
	5-LOX	Hepatocyte	5-LOX/LTB4	LTB4 ↑	liver regeneration ↑	^477,478^
	CYP epoxygenases	Endothelial cell	CYP epoxygenases/EET	EET ↑	liver regeneration ↑	^481^
	MAGL	Hepatocyte	MAGL/Eicosanoid	PGE2 ↑ , TXA2 ↑	liver regeneration ↑	^484^
Cholesterol	HMGCR	Hepatocyte	HMGCR/Cholesterol/Insulin receptor/Ca2+ signals, HMGCR/Cholesterol/Insulin receptor/AKT	cholesterol synthesis ↑ glucose uptake ↑	liver regeneration ↑	^489,490^
	Caveolin-1	Hepatocyte, HSC, KC and endothelial cell	Cholesterol/Caveolin-1	oxidation of fatty acids ↑, ketogenesis ↑, mitochondrial respiration ↑, bile acid signaling transduction	liver regeneration ↑	^491,493–495^
	LXR	Hepatocyte	LXR/ABCA1	cholesterol efflux	liver regeneration ↓	^487^
	TLR	Macrophage	LXR/ABCA1/TLR4/Myd88	cholesterol efflux	liver regeneration ↓	^498–502^
Bile acids	FXR	Hepatocyte	BA/FXR/INSIG-2/HMGCR BA/FXR/FGF15/CYP7A1 FGF15/FGFR4/GSK-3 FGF15/FGFR4/PGC-1α	BA synthesis ↓ cholesterol synthesis ↓ glycogen synthesis ↓ gluconeogenesis ↑	liver regeneration ↑	^524–526,528,529^
Retinoid	Retinol droplets	HSC	-	-	liver regeneration ↑	^543^
Sphingolipids	SPT	Hepatocyte	Wnt/β-catenin	sphingomyelin synthesis ↑	liver regeneration ↑	^269,550^
Lipid ligands	PPARα	Hepatocyte	PPARα/SPT, mTORC2/GluCer/PPARα	sphingomyelin synthesis ↑, oxidation of fatty acids ↑	liver regeneration ↑	^557–559^
	PPARβ	Hepatocyte	PPARβ/AKT, PPARβ/E2F	glycolysis ↑, fatty acids synthesis ↑	liver regeneration ↑	^562^
	PPARγ	Hepatocyte	PPARγ/MGAT1	triglyceride synthesis ↑, glucose tolerance ↓	liver regeneration ↓	^565^
Amino acids	GCN5L1	Hepatocyte	GCN5L1/ERK/FOXO1/PEPCK + G6P GCN5L1/GLS/α-KG	gluconeogenesis ↑, glutaminolysis ↓	liver regeneration ↓	^575,576^
	Catecholamines	Hepatocyte	GP, FBPase, PEPCK, CPT	glycogenolysis ↑, gluconeogenesis ↑, oxidation of fatty acids ↑	liver regeneration ↑	^182^
TCA cycle	Succinate	M1 macrophage	Succinate/HIF-1α	glycolysis ↑	liver regeneration ↓	^591^
	4-octyl itaconate	M2 macrophage	GAPDH	glycolysis ↓	liver regeneration ↑	^598^
ATP	MCJ	Hepatocyte, macrophage	Complex I	ATP ↓, insulin sensitivity ↓, ROS ↑	liver regeneration ↓	^607,613^

### Glucose metabolism

The liver is involved in maintaining glucose homeostasis. Extrahepatic and intrahepatic glycometabolism are crucial for the energy supply and production of anabolic molecules during liver repair and regeneration. Metabolomics has revealed significant changes in metabolites before and after hepatectomy.^387,389^ Although the exact mechanisms driving these effects remain to be fully clarified, several studies have highlighted the importance of hypoglycemia in triggering the process of liver regeneration following acute liver injury. Hypoglycemia is one of the first manifestations of metabolic reprogramming during the early phases of liver regeneration after PHx, which is likely attributable to a marked reduction in hepatic glycogen stores and gluconeogenic capacity in the metabolic stress response.^387,390^ A metabolite analysis revealed that gluconeogenesis is markedly suppressed in the priming regenerative phase.^388^ In parallel with its affecting metabolic pathways, hypoglycemia also plays a role in regulating liver repair and regeneration. Post-PH hypoglycemia can promote Cyclin D1 expression to induce early G1 progression.

Interestingly, glucose supplementation can inhibit hepatic regeneration by increasing the p21 and p27 expression, while simultaneously reducing the Forkhead box protein M1 (FOXM1) expression. Previous studies reveal FOXM1 suppresses p21 expression, and its downregulation impairs liver regeneration. Reciprocally, p21 negatively regulates FOXM1, creating a feedback loop that controls cell proliferation.^391–394^ Although early hypoglycemia is favorable for stimulating liver regeneration, sustained and rapid untreated hypoglycemia can lead to death.^395^

**Fig. 5:**
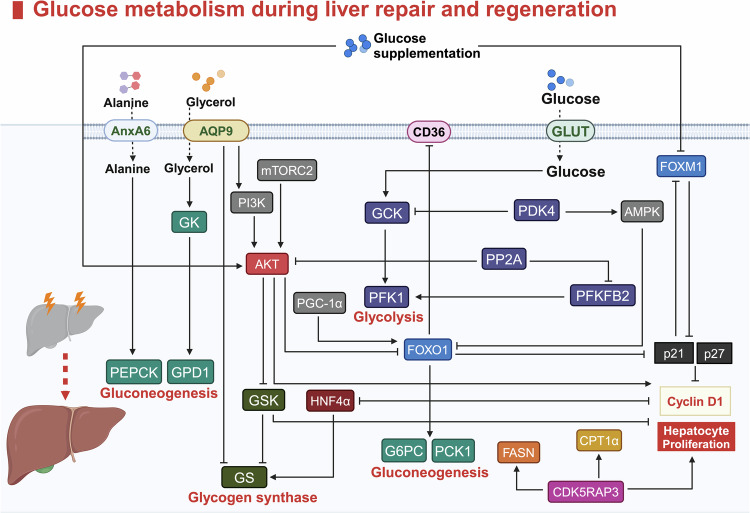
Glucose metabolism during liver repair and regeneration. A complex metabolic network regulates liver regeneration via gluconeogenesis, glycogen synthesis, and glycolysis. The arrows indicate upregulation or activation, and the T-arrows indicate downregulation or inhibition. AnxA6 annexin A6, AQP9 aquaporin-9, GLUT glucose transporter type, PEPCK phosphoenolpyruvate carboxykinase, GK glycerol kinase, GPD1 glycerol-3-phosphate dehydrogenase 1, GSK, glycogen synthase kinase, GS glycogen synthase, GCK glucokinase, PFK1 phosphofructokinase 1, PDK4 pyruvate dehydrogenase lipoamide kinase isozyme 4, PP2A, protein phosphatase 2, FOXM1, forkhead box protein M1, PFKFB2 6-phosphofructo-2-kinase/fructose-2,6-bisphosphatase-2, FOXO1 forkhead box protein O1, HNF4α hepatocyte nuclear factor 4α, G6PC glucose-6-phosphatase catalytic subunit, PCK1 phosphoenolpyruvate carboxykinase 1, FASN fatty acid synthase, CPT1α carnitine palmitoyltransferase 1α, CDK5RAP3 Cyclin-dependent kinase 5 regulatory subunit associated protein 3. The figure was generated with BioRender (https://biorender.com)

The remaining liver undergoes metabolic reprogramming after injury, adjusting the balance between gluconeogenesis, glycogen synthesis, and glycolysis to meet the substantial energy, substrate, and signaling demands required for repair and regeneration (Fig. 5).

#### Gluconeogenesis

The liver is the central organ involved in gluconeogenesis. When glycogen is depleted, alanine, lactate and glycerol are transported to the liver and serve as precursors for gluconeogenesis. The key transcription factors and substrates related to gluconeogenesis have been shown to be involved in liver regeneration. The expression of AKT, a set of three serine/threonine-specific protein kinases, is high in the liver. Several studies have highlighted the importance of the PI3K/PDK1/AKT pathway in cell growth.^42,396,397^ The transcription factor FOXO1 is a target of AKT during liver regeneration. Additional KO of FOXO1 in AKT-deficient mice restores Cyclin D1 expression and reverses the impaired capacity for liver regeneration after PHx.^398,399^ Recent findings have uncovered the upstream mechanism by which mTORC2 is involved in partially regulating AKT1/2 kinase activity.^400,401^ Coupled with its role in regulating cell proliferation, AKT signaling is involved in regulating glucose homeostasis and lipid metabolism.^402–405^ The liver-specific deletion of both AKT1 and AKT2 inhibits hepatocyte proliferation along with glucose intolerance and insulin resistance. Further research has revealed that, with the assistance of the coactivator PGC-1α, FOXO1 regulates glycogenolysis and gluconeogenesis by modulating the expression of G6PC and PCK1.^295,402,406,407^

Alanine is an important substrate for gluconeogenesis. Annexin A6 (AnxA6) is a Ca2^+^-dependent phospholipid-binding protein that regulates alanine uptake in the liver. A lack of AnxA6 compromises alanine-dependent gluconeogenesis and impairs liver regeneration due to insufficient energy via the inhibition of phosphoenolpyruvate carboxykinase (PEPCK).^408^ Aquaporin-9 (AQP9) is responsible for transporting glycerol, a key substrate in gluconeogenesis, from sinusoidal blood. Deficiency in AQP9 impairs glycerol uptake, which subsequently inhibits gluconeogenesis by reducing the levels of glycerol kinase (GK) and glycerol-3-phosphate dehydrogenase 1 (GPD1). Additionally, AQP9 deficiency leads to impaired glycogen synthesis, evidenced by increased levels of phosphorylated glycogen synthase (GS). The activity of the PI3K/AKT signaling pathway is also significantly decreased in AQP9 KO hepatocytes. As a result, AQP9 deficiency delays hepatocyte proliferation and increases mortality following liver injury.^409^ AQP9 deficiency not only disrupts glucose metabolism, but also disrupts the transport of H_2_O_2_, a crucial signal in the liver’s proliferative phase. Impaired H_2_O_2_ transport leads to intracellular H_2_O_2_ overload, increasing oxidative stress and exacerbating liver injury during regeneration.^409–411^

#### Glycogen synthesis

Glycogen synthesis, catalyzed by GS, is tightly regulated by complex mechanisms. GSK-3, a serine/threonine protein kinase, was originally identified for its ability to phosphorylate and inhibit GS. Suppressing GSK-3 activity increases glycogen synthesis, enhances insulin sensitivity, and improves glucose homeostasis in the liver.^388^ However, the pharmacological or genetic inhibition of GSK-3 has been shown to impair liver regeneration.^412–414^ Consistent with this finding, delaying PH-induced hypoglycemia slows liver regeneration and disrupts energy reserves through increased phosphorylation of GSK-3 via AKT.^388,391,415^

Factors influencing glycogen synthesis also play a role in liver repair and regeneration. Cyclin-dependent kinase 5 regulatory subunit associated protein 3 (CDK5RAP3) has been shown to bind and activate cyclin-dependent kinase 5.^416^ Liver-specific CDK5RAP3 KO significantly delays liver regeneration and reduces glycogen synthesis. Additionally, the removal of CDK5RAP3 decreases the expression of key metabolic genes, including carnitine palmitoyltransferase 1α (*Cpt1α*), involved in β-oxidation, and fatty acid synthase (*Fasn*), which is related to fatty acid synthesis.^417^ Since glucose metabolism and lipid oxidation provide essential energy sources after partial hepatectomy, these findings suggest a potential link between liver regeneration and glucose/lipid metabolism.^388,418^

These findings underscore the intricate relationship between regeneration and metabolic reprogramming following injury. Recent studies have revealed that the mutual inhibition of Cyclin D1 and hepatocyte nuclear factor 4α (HNF4α) harmonizes to regulate the progression of the cell cycle and metabolism within the liver.^419–422^ Cyclin D1 restrains glucose uptake and glycogen synthesis via HNF4α-regulated metabolic adaptation. In contrast, HNF4α deletion in the liver induces Cyclin D1 expression and hepatocyte proliferation.^421^ The coordination between cell cycle regulation and metabolic adaptation redirects intracellular resources to meet the demands of proliferation during liver regeneration.

#### Glycolysis

The precise role of glycolysis in liver regeneration remains inconclusive, depending on the regulatory context and the specific stage of the regenerative process. Understanding the dual role of glycolysis in liver regeneration requires a review of its key metabolic steps. Glucose uptake is facilitated by plasma membrane GLUT, followed by phosphorylation by glucokinase (GCK) to generate glucose 6-phosphate (G6P). This intermediate is converted to fructose 6-phosphate (F6P) and subsequently to fructose 1,6-bisphosphate (FBP) by PFK1, a critical regulatory step in the glycolytic pathway.^423^

Protein phosphatase 2 (PP2A), a serine/threonine phosphatase, is markedly upregulated during the termination of liver regeneration and participates in this process through the AKT/GSK-3β/Cyclin D1 pathway.^424,425^ Further analysis of glycolytic flux revealed that the catalytic subunit of PP2A inhibits hepatic glycolysis via 6-phosphofructo-2-kinase/fructose-2,6-bisphosphatase-2 (PFKFB2), contributing to the termination of liver regeneration.^424,426^ This provides important insights into the metabolic reprogramming associated with liver regeneration, and models of chronic liver injury similarly offer critical insights into the role of glycolysis in this process. Liu et al. reported that FXR activation prevents the proliferation of Sox9^+^ hepatocytes by increasing glycolysis and inhibiting oxidative phosphorylation (OXPHOS) in a CCl_4_-induced model.^427^

Interestingly, another study has provided indirect evidence, offering further clues into the metabolic pathways involved in liver regeneration. Pyruvate dehydrogenase lipoamide kinase isozyme 4 (PDK4) serves as a crucial enzyme, and loss of PDK4 drives the proliferative response in the remnant liver tissue.^428^ PDK4 deficiency enhances hepatic glycolysis by increasing the GCK level and improving insulin sensitivity during liver regeneration. Alongside the enhancing hepatic insulin/AKT signaling, PDK4 inhibition also activates the lipid AMPK/FOXO1/CD36 regulatory axis to promote liver regeneration by increasing fatty acid uptake.^428^

While hepatocyte metabolic reprogramming plays a crucial role in liver regeneration, the metabolic shifts in non-parenchymal cells are equally important and warrant further exploration. Altered cellular metabolism is an important characteristic of macrophage polarization. M1 macrophages exhibit a proinflammatory phenotype characterized by increased glycolysis and impaired mitochondrial OXPHOS.^429–433^ The mechanism of enhanced glycolysis involves the activation of pyruvate kinase isoenzyme M2 (PKM2), which triggers downstream inflammatory factors in a hypoxia-inducible factor 1α (HIF-1α)-dependent manner.^434–436^ Glycogen synthesized from glycolysis-derived G6P also repolarizes macrophages to an inflammatory phenotype via the pentose phosphate pathway and UDPG-P2Y14 signaling pathway.^437^ M2 macrophages are thought to be associated with tissue repair and cell proliferation and exhibit an intact tricarboxylic acid (TCA) cycle with increased OXPHOS and fatty acid oxidation.^429,431^ Switching hepatic macrophage polarization to the M2 phenotype is associated with decreased glycolysis and increased mitochondrial oxidative phosphorylation.^426,438–441^ FOXO1 deficiency has been shown to drive macrophages towards an M2 phenotype. Transcriptomic and proteomic analyses further highlight FOXO1 as a crucial link between glycolytic metabolism and macrophage polarization.^442^ The metabolic reprogramming of macrophages to regulate inflammatory conditions provides novel insights into potential treatments for liver regeneration.

### Lipid metabolism

**Fig. 6:**
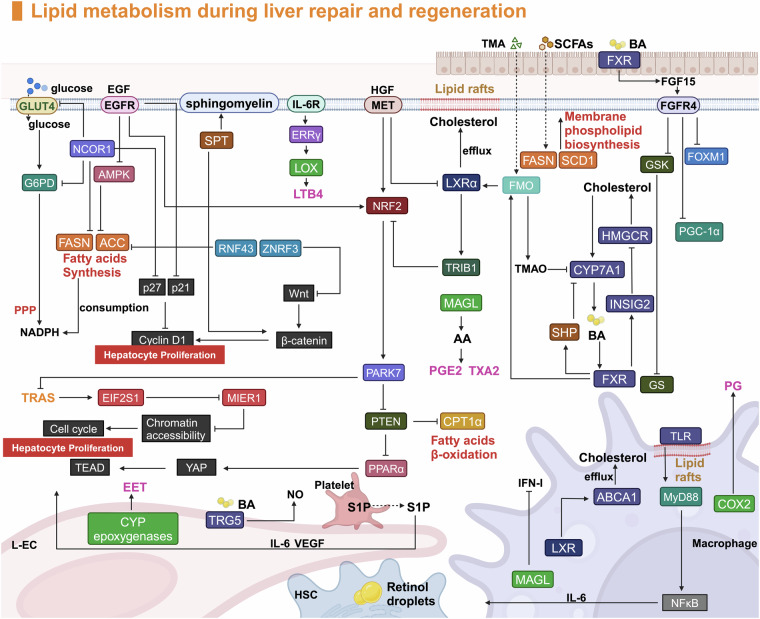
Lipid metabolism during liver repair and regeneration. Alterations in lipid metabolism during liver repair and regeneration. The arrows indicate upregulation or activation, and the T-arrows indicate downregulation or inhibition. TRAS transient regeneration-associated steatosis, TMA trimethylamine, TMAO trimethylamine N-oxide, FMO3 flavin-containing monooxygenase 3, BA bile acid, SCFA short-chain fatty acid, FGF fibroblast growth factor, FGFR4 fibroblast growth factor receptor 4, GLUT4 glucose transporter type 4, G6PD glucose-6-phosphate dehydrogenase, NADPH nicotinamide adenine dinucleotide phosphate, PPP pentose phosphate pathway, NCOR1 nuclear receptor corepressor 1, AMPK AMP-activated protein kinase, FASN fatty acid synthase, ACC acetyl-CoA carboxylase, SCD1 stearoyl-CoA desaturase 1, CPT1α carnitine palmitoyltransferase 1α, TEAD TEA domain family member, ZNRF3 Zinc and Ring Finger 3, RNF43 Ring Finger Protein 43, EGF epidermal growth factor, EGFR epidermal growth factor receptor, IL-6 interleukin-6, IL-6R, IL-6 receptor, VEGF vascular endothelial growth factor, MET mesenchymal-epithelial transition factor, HGF hepatocyte growth factor, SREBF1 sterol regulatory element-binding transcription factor 1, SPT serine palmitoyltransferase, ERRγ estrogen-related receptor γ, LOX lipoxygenase, COX cyclooxygenase, NRF2nuclear factor erythroid 2-related factor 2, ROS reactive oxygen species, LXRα liver X receptor α, TRIB1 tribbles homolog 1, MAGL monoacylglycerol lipase, PARK7 parkinsonism‐associated deglycase 7, PTEN phosphatase and tensin homolog, MIER1 mesoderm induction early response 1, EIF2S eukaryotic translation initiation factor 2 subunit, EET epoxyeicosatrienoic acid, TXA2 thromboxane A2, LTB4 leukotriene B4, PG prostaglandin, PGE2 prostaglandin E2, TRG5 G protein-coupled bile acid receptor 5, CYP cytochrome P450, NO nitric oxide, S1P sphingosine 1-phosphate, CYP7A1 cholesterol 7 alpha-hydroxylase, FXR farnesoid X receptor, HMGCR HMG-CoA reductase, INSIG2 insulin induced gene 2, SHP small heterodimer partner, GSK glycogen synthase kinase, GS glycogen synthase, FOXM1 forkhead box protein M1, PGC‐1α peroxisome proliferator‐activated receptor‐γ coactivator‐1α, PEPCK phosphoenolpyruvate carboxykinase, TLR Toll-like receptor, ABCA1 ATP-binding cassette transporter-A1, PPARα peroxisome proliferator-activated receptor α. The figure was generated with BioRender (https://biorender.com)

Considerable evidence of alterations in lipid metabolism is observed in both resection- and toxin-induced liver regeneration.^443^ Lipids that accumulate during regeneration not only serve as the energy supply and substrates for membrane synthesis but also directly contribute to regulating the downstream production of metabolites to coordinate cell proliferation via transcriptional or epigenetic mechanisms.^443^ Hepatic lipid remodeling results in a favorable metabolic and inflammatory environment to promote the compensatory proliferation of hepatocytes in subjects with nonalcoholic steatohepatitis (NASH).^387^ Acute lipid accumulation in the liver induces acute stress and inhibits mesoderm induction early response 1 (*Mier1*) translation via the eukaryotic translation initiation factor 2 subunit (EIF2S) signaling pathway.^444,445^ Reduced expression of MIER1, a key epigenetic regulator, increases chromatin accessibility and especially cell cycle-related gene transcription after surgical resection, ultimately accelerating liver regeneration.^445,446^ Although the mechanisms related to lipid metabolism in liver regeneration have not been fully elucidated, several potential targets deserve further consideration (Fig. 6).

#### Fatty acids

Fatty acids are the primary source of energy for liver regeneration. Synthesis and oxidation are crucial processes for maintaining the homeostasis of fatty acid metabolism in the liver.^447^ Here, we discuss several candidates linking fatty acid metabolism and liver repair and regeneration.

##### Fatty acid synthesis

Many studies indicate that appropriate, transient regeneration-associated steatosis (TRAS) is a universal feature of regenerating livers, recognized as an essential process for the initial phases of liver regeneration.^418,443,448^ Nuclear receptor corepressor 1 (*Ncor1*), a comprehensive nuclear receptor inhibitory gene, regulates the crosstalk among several nuclear proteins related to metabolism.^449–451^ Hepatic NCOR1 deficiency results in increased hepatocyte proliferation and downregulated P27 expression following PHx. NCOR1 deficiency triggers lipid synthesis in hepatocytes by increasing FASN and acetyl-CoA carboxylase (ACC) expression.^451^ Further research revealed that NCOR1 KO increases glucose flux via the upregulation of GLUT4 and glucose-6-phosphate dehydrogenase (G6PD) expression. Increased glucose intake and mitochondrial activity meet the demands for increased substrate and ATP consumption for de novo fatty acid synthesis. The accumulation of these lipids can provide more energy via fatty acid oxidation for cell cycle entry.^451^ In NCOR1 KO mice, nicotinamide adenine dinucleotide phosphate (NADPH) consumption for de novo fatty acid synthesis can occur, and redox homeostasis can be maintained by activating the G6PD-dependent pentose phosphate pathway (PPP).^451^

Inhibition of the EGFR leads to AMPK activation, which phosphorylates ACC and suppresses regeneration-associated lipid accumulation.^374,452,453^ Additionally, EGFR inhibition in a NAFLD-associated steatosis model similarly results in reduced expression of core lipid metabolism enzymes, potentially regulated by the transcription factors ChREBP, SREBF1, PPARγ, and HNF4α. This is accompanied by a reduction in Cyclin D1 expression, further implicating a connection between lipid metabolism and cell cycle regulation.^452,454^

Moderate fatty acid synthesis is essential not only for initiating hepatic regeneration but also for properly terminating the regenerative process. RNF43 and ZNRF3 are two negative feedback regulators of Wnt signaling.^255,455^ Hepatocyte-specific loss of RNF43/ZNRF3 disrupts lipid metabolism, leading to increased unsaturated fatty acid biosynthesis and altered lipid zonation.^264,265^ Interestingly, in *Rnf43/Znrf3* mutants, hepatocytes remain in a proliferative state after regeneration is initiated, failing to properly halt the process. This failure to halt regeneration leads to unchecked hepatocyte proliferation, ultimately predisposing the mice to liver cancer.^264^

##### Fatty acid oxidation

The inhibition of ß-oxidation is also associated with the inhibition of the regenerative response of the liver.^448,456–459^ A large amount of stored fat can fulfill the energy and substrate demands for liver regeneration. The energy provided by lipids during reparative processes is produced mainly by the oxidation of fatty acids.

EGF and HGF regulate TRAS by modulating transcription factors involved in lipolysis and fatty acid biosynthesis through their respective receptors EGFR and c-Met following PHx.^374,460^. Impaired lipid metabolism mediated by the elimination of EGFR can be compensated by MET signaling without abnormal liver regeneration.^374,454^ Interestingly, subsequent studies revealed, nuclear factor erythroid 2-related factor 2 (NRF2) expression can be induced by HGF and EGF, activating the parkinsonism-associated deglycase (*Park*) gene.^459^ PARK7 deficiency hinders fatty acid β-oxidation and ATP production by inhibiting the expression of PPARα and carnitine palmitoyltransferase 1α (CPT1α) in a PTEN-dependent manner. This ultimately prolongs TRAS and delays liver regeneration after PHx.^418,443,459^

HGF also affects intracellular redox balance, which is crucial for liver regeneration. HGF modulates ROS levels and glutathione (GSH) production by displacing liver X receptor (LXRα) from the Tribbles homolog 1 (*Trib1*) promoter. Subsequently, TRIB1 regulates redox balance and liver regeneration by controlling NRF2 activity.^461^ During regeneration, TRIB1 downregulation permits NRF2 nuclear translocation, activating antioxidant response and fostering a conducive environment for hepatocyte proliferation.^461–464^ Notably, recent studies further support the pivotal role of NRF2 in intracellular metabolic reprogramming during hepatocyte proliferation, demonstrating its involvement in promoting glycolysis, nucleic acid synthesis, the oxidative pentose phosphate pathway (PPP), and NAD^+^/NADH synthesis, while downregulating OXPHOS.^43^ These studies provide a potential mechanism by which EGFR/MET signaling orchestrates cellular metabolism to affect liver regeneration.^465^

However, lipid overload and prolonged lipid accumulation can induce hepatocyte apoptosis and increase endoplasmic reticulum (ER) stress. In both clinical and experimental settings, pre-existing steatosis or excessive transient steatosis leads to a marked impairment of the regeneration capacity.^466–468^

##### Short-chain fatty acids

Short-chain fatty acids (SCFAs), major gut microbial metabolites, have multiple physiological functions. Acetate, propionate, and butyrate are the most abundant SCFAs in the gut.^469^ SCFAs are thought to contribute to liver regeneration though the gut‒liver axis.

Phospholipid biosynthesis is critical for membrane formation during hepatocyte replication. Isotope labeling experiments have demonstrated that bacterially derived SCFAs contribute to both phospholipid biosynthesis and hepatocyte proliferation following PHx.^470^ Mechanically, SCFAs induce fatty acid biosynthesis enzymes, including stearoyl-CoA desaturase 1 (SCD1) and FASN.^470^ Notably, SCD1 expression is significantly elevated in hyperproliferative regions compared to atrophic areas in patients after ALPPS.^470^

SCFAs also contribute to regulating immunometabolic homeostasis during liver regeneration.^471^ SCFAs enhance gut barrier integrity, reducing LPS translocation and minimizing the transfer of proinflammatory substances to the liver, thereby curbing subsequent inflammatory responses.^471,472^ For instance, acetate can regulate inflammatory responses by affecting lipid accumulation in macrophages in a dose-dependent manner.^473^ The regulatory mechanisms of these inflammatory responses and metabolic remodeling offer potential therapeutic targets for mitigating liver injury and enhancing liver repair and regeneration.

##### Eicosanoids

Eicosanoids, derived from polyunsaturated fatty acids, play a key role in inflammatory processes and immune modulation. Previous studies have shown that eicosanoids, such as prostaglandins (PGs), leukotrienes (LTs), thromboxane A2 (TXA2), and epoxyeicosatrienoic acids (EETs), are critical regulators of liver regeneration.^474^

Following PHx, the levels of PGs, notably PGE2 and PGF2α, are elevated, primarily due to increased synthesis from KCs and hepatocytes. Inhibition of cyclooxygenase 2 (COX2) markedly diminishes PG production, thereby impeding liver regeneration.^475,476^ PG acts in concert with NO, and the simultaneous inhibition of nitric oxide synthase-2 (NOS2) and COX2 further hinders this process. Importantly, the administration of excess exogenous NO or PG has been shown to reverse these inhibitory effects, underscoring their critical roles in facilitating liver recovery.^474^

In the early stages of liver regeneration, increases in 5-lipoxygenase (5-LOX) levels and its product leukotriene B4 (LTB4) are noted. The administration of 5-LOX or LTB4 inhibitors significantly delays hepatocyte proliferation.^477^ Further investigations have demonstrated that LOX expression is modulated by the nuclear receptor estrogen-related receptor γ (ERRγ) in hepatocytes upon IL-6 stimulation.^478^ Notably, reduced hepatic LTB4 levels correlate with diminished recruitment of neutrophils and macrophages in response to the 5-LOX inhibitor zileuton.^479^ The recruitment of these immune cells may also play a critical role in modulating repair and regeneration.

Liver repair and regeneration are, in part, regulated by angiogenesis.^203,480^ Epoxyeicosatrienoic acids (EETs), metabolites of arachidonic acid produced by cytochrome P450 (CYP) epoxygenases, are predominantly synthesized in endothelial cells.^481^ Panigrahy et al. reported that a substantial rise in endothelial-derived EETs supports the growth of the regenerating liver. This pro-regenerative effect is mediated via a VEGF-dependent pathway, highlighting the critical role of EETs in liver recovery.^481^

Monoacylglycerol lipase (MAGL) is an enzyme involved in the degradation of monoacylglycerols and the production of eicosanoids.^482,483^ Monoacylglycerol lipase (MAGL) plays a key role in degrading monoacylglycerols and producing eicosanoids. Hepatocyte-specific deletion of *Magl* impairs liver regeneration, attributed to reduced levels of arachidonic acid-derived metabolites, such as PGE2 and TXA2.^484^ Additionally, MAGL KO in myeloid cells results in defective regenerative capacity, linked to macrophage reprogramming toward an IFN-I pathway profile. However, this impaired regeneration can be restored through in vivo blockade of the IFN-I pathway.^484^

#### Cholesterol

Cholesterol is a fundamental constituent of biological membranes, essential for maintaining the structural integrity of the lipid bilayer, thereby influencing key membrane functions such as material exchange, ligand recognition, and signal transduction.^485,486^ Increase in hepatic cholesterol levels requires the suppression of LXR transcriptional pathways to preserve the intracellular cholesterol necessary for effective hepatocyte regeneration.^487,488^

Within these membranes, lipid rafts, which are specialized microdomains rich in cholesterol, glycosphingolipids, and protein receptors, play a pivotal role in regulating liver regeneration by coordinating signaling pathways. As a result, the integrity and composition of these lipid rafts are critical for orchestrating effective regenerative responses.^489^ A reduction in cholesterol in the plasma membrane by the HMG-CoA reductase (HMGCR) inhibitor lovastatin leads to the impaired distribution of insulin receptors in lipid rafts, the aberrant transduction of nuclear Ca^2+^ signals in hepatocytes, and delayed liver regeneration.^489,490^

Caveolae, a specialized subtype of lipid rafts, are highly dependent on cholesterol for their formation and are moderately expressed on hepatocytes, HSCs, KCs, and LSECs. The primary structural proteins of caveolae, known as caveolins, have a strong affinity for cholesterol and play key roles in several metabolic processes, including glucose homeostasis, bile acid signaling, lipid transport, ketogenesis, and mitochondrial respiration.^479,491,492^ Caveolin-1 deficiency disrupts lipid accumulation and impairs liver regeneration, although this defect can be rescued by exogenous glucose supplementation.^493,494^ Metabolic analysis in Caveolin-1 KO mice indicates that the regenerative capacity is maintained through compensatory mechanisms, including enhanced aerobic glycolysis, overactive lipogenesis, and increased activity of the pentose phosphate pathway.^495^ Beyond its role on the plasma membrane, caveolae have been identified on peroxisomal membranes. This suggests that Caveolin-1 may facilitate the transport of lipid metabolites between peroxisomes and the cytosol, potentially linking these processes to the metabolic requirements of liver regeneration.^496^

Accumulated cholesterol also activates the immune system and initiates an inflammatory.^488,497^ Dietary cholesterol reprograms lipid metabolism and polarization gene expression in hepatic macrophages, driving their phenotypic shift toward tissue repair and regeneration.^498^ In macrophages, LXRα, which is highly expressed, serves as a crucial link between cholesterol metabolism and inflammatory responses.^499^ It regulates cholesterol efflux by inducing the transcription of ATP-binding cassette transporter-A1 (ABCA1), thereby redistributing membrane cholesterol and disrupting lipid rafts, which TLR complexes and hinders the recruitment of MyD88.^500–502^ The TLR/MyD88 pathway in macrophages plays a key role in liver regeneration through the activation of NFκB and the production of IL-6, further linking metabolic reprogramming and inflammation.^106^ Selective activation of LXRα in macrophages, without affecting hepatocytes, suggests the potential for targeting LXR in macrophage-mediated liver regeneration.^503^

Neutrophils, another key player in liver regeneration, are also influenced by cholesterol metabolism. Following liver injury, cholesterol secreted by hepatocytes activates ERRα in neutrophils, triggering the release of HGF to promote hepatocyte proliferation. This cholesterol–ERRα–HGF axis highlights cholesterol’s role in mediating neutrophil-hepatocyte cross-talk to drive liver repair and regeneration.^161^

Reshaping cholesterol metabolism is a crucial step in the immune response to T-cell activation. Cholesterol regulates metabolic programming that supports T-cell differentiation and activation.^504,505^ Defective cholesterol metabolism, such as in LXRβ-deficient T cells, impairs their proliferative capacity following activation.^505^ Moreover, LXR regulates membrane lipid composition, influencing glycosphingolipid levels and cellular functions such as IL-4 production.^504^ Taken together, these findings highlight the broader relevance of cholesterol metabolism in immune regulation.

#### Bile acids

The circulation of bile acids (BAs) plays a crucial regulatory role in liver repair and regeneration through the liver‒gut axis,^506,507^ with impaired regenerative capacity observed following external biliary drainage.^507–509^

An immediate overload of BAs in the systemic blood and liver occurs within hours after PHx.^510–512^ In fact, BAs act as crucial metabolic signals that promote hepatocyte proliferation by activating a range of nuclear and membrane receptors. Among these, Takeda G protein-coupled receptor 5 (TRG5) and FXR are the most extensively studied. TGR5 is highly expressed in BECs, KCs and LSECs. Liver regeneration is significantly impaired in TGR5 KO mice compared to wild-type controls, highlighting the protective role of TGR5 in this process.^513^ However, excessive BAs can be detrimental, leading to hepatocyte death.^514^ TGR5 helps mitigate BA overload, regulating their composition and concentration to create a more favorable environment for liver regeneration.^375,515^ Through TGR5 activation, secondary BAs also modulate the hepatic inflammatory response by inhibiting LPS-induced cytokine expression in macrophages, thereby reducing hepatocyte necrosis and contributing to liver repair after PHx.^516^ Furthermore, TGR5 promotes the polarization of macrophages from a pro-inflammatory M1 state to an anti-inflammatory M2 state, positioning certain bile acids as intrinsic regulators of macrophage polarization and liver repair.^516^ In parallel, TGR5 activation in LSECs triggers NO production, which enhances hepatocyte sensitivity to HGF, further supporting liver regeneration.^517^

Studies have also highlighted the interactions between BAs and extrahepatic organs during liver regeneration. Watanabe et al. reported that certain types of secondary BAs can activate TGR5, leading to the synthesis of active thyroid hormones in brown adipose tissue and skeletal muscle.^518^ The activation of thyroid hormone β receptors subsequently stimulates mitogenesis and hepatocyte proliferation by modulating the Wnt/β-catenin pathway, both in the normal liver and in the remnant liver after resection.^519,520^ Additionally, TGR5 activation in colonic L cells promotes the secretion of glucagon-like peptide-1, which enhances insulin production.^521^ Elevated hepatic insulin/AKT signaling supports liver regeneration by driving the synthesis of proteins, lipids, and glycogen.^42,428^

Another key nuclear receptor is FXR, also known as the bile acid receptor, which is activated by bile acids in both hepatocytes and enterocytes and plays a critical role in maintaining BA homeostasis. FXR signaling is essential for normal liver regeneration.^522^ While hepatocyte- or intestine-specific deletion of FXR delays liver regeneration without causing mortality, global FXR KO mice exhibit severely impaired regeneration and high mortality rates.^523^ FXR plays a dual role in stimulating liver regeneration. On one hand, FXR protects the liver from bile acid overload by repressing bile salt synthesis through the inhibition of CYP7A1, a process mediated by the induction of small heterodimer partner (SHP).^524^ It also reduces cholesterol synthesis by inducing the expression of hepatic insulin induced gene 2 (INSIG2), which inhibits HMGCR.^525,526^ On the other hand, FXR activation in hepatocytes promotes liver regeneration by inducing the expression of the cell cycle protein FOXM1.^512,522,523^

FXR signaling in both the liver and intestines coordinates liver regeneration via the gut‒liver axis. FXR activation in enterocytes induces FGF15/19, which activates fibroblast growth factor receptor 4 (FGFR4) on hepatocytes to stimulate regeneration.^512,527^ This mechanism also influences energy homeostasis by inhibiting hepatic glycogen synthesis through GSK-3 and promoting gluconeogenesis via PGC‐1α.^528,529^ Moreover, both FGFR4/β-Klotho and Wnt signaling are highly active in Zone 3 of the liver. FGF19 can synergize with Wnt signaling to accelerate mitogenic activity, thereby enhancing regeneration in vivo.^530^

The composition and hydrophobicity of BAs have been shown to modulate liver regeneration.^531–533^ Moreover, maintaining an appropriate bile acid-to-cholesterol ratio is essential for balancing proliferation and fibrosis after hepatotoxin-induced damage,^534^ further highlighting the critical role of BA homeostasis during injury repair. Emerging evidence highlights the role of trimethylamine N-oxide (TMAO) in regulating bile acid metabolism and liver regeneration. TMAO influences the bile acid pool by downregulating the expression of key bile acid synthesis enzymes CYP7A1 and CYP27A1.^535^ Further studies revealed that the trimethylamine (TMA)/flavin-containing monooxygenase 3 (FMO3)/TMAO pathway is likely to regulate the cholesterol balance and inflammatory response via both the LXR and the FXR signaling pathways.^536,537^ Moreover, TMAO affects liver physiology by modulating macrophage metabolic remodeling, reducing matrix metalloproteinase 12 levels, which impairs macrophage migration and delays liver regeneration following AILI.^536,538^

#### Retinoids

The majority of retinoids are stored in lipid droplets within HSCs.^539^ A significant decrease in cytoplasmic retinoid droplet storage has been observed during liver regeneration.^540,541^ The depletion of hepatic retinoid stores impairs the initial recovery of liver mass, suggesting a link between retinoid metabolism in HSCs and hepatocyte proliferation.^539^ Retinol release and the loss of lipid droplets appear to be crucial for HSC activation, as free retinol released by HSCs is absorbed by hepatocytes in a contact-dependent manner.^542^ Verónica et al. reported that endogenous retinol in HSCs supports liver regeneration by serving as a substrate for liver alcohol dehydrogenase (ADH).^543^ Additionally, all-trans retinoic acid, a derivative of retinoids, modulates cell cycle progression by regulating Cyclin-Cdk complexes, thereby triggering hepatocyte mitosis.^544–546^

Activated HSCs undergo metabolic shifts in carbohydrate metabolism, mitochondrial function, and glutaminolysis to support proliferation.^547,548^ While not fully understood, these pathways offer potential targets for therapies to enhance hepatocyte regeneration.

#### Sphingolipids

Sphingolipids, a class of lipids with a sphingoid base backbone, are integral to the plasma membrane lipid bilayer, with sphingomyelin being a key component of membrane rafts.^549^

Serine palmitoyltransferase (SPT) is critical for sphingomyelin biosynthesis. Liver-specific deficiency of *Sptlc2*, which encodes the essential SPTLC2 subunit of SPT, impairs hepatocyte polarity and inhibits liver regeneration.^269,550^ Mechanistically, SPTLC2 KO leads to cadherin degradation and disrupts β-catenin distribution.^269^ The involvement of sphingolipids in the Wnt/β-catenin pathway has been well-documented,^551,552^ with delayed liver regeneration in response to various liver injury.^243,553,554^ Notably, impaired regeneration due to β-catenin KO in hepatocytes is eventually compensated by insulin/mTORC1 signaling activation. However, dual inhibition of β-catenin and mTORC1 completely blocks liver regeneration and results in early lethality.^272^

Sphingolipid-derived metabolites, such as S1P, promote liver regeneration by driving proregenerative vascular remodeling and the production of inflammatory mediators in an LSEC-dependent manner.^225,555^ The transfer of neutral ceramidase and sphingosine kinase 2 (SK2) to hepatocyte exosomes has been shown to enhance S1P synthesis in target hepatocytes.^556^ Kawasaki et al. demonstrated that direct contact between LSECs and platelets triggers S1P secretion from platelets, which subsequently induces LSECs to produce IL-6 and VEGF, modulating DNA synthesis in hepatocytes.^224^

#### Lipid-derived PPAR ligands

PPARs are involved in modulating multiple biological processes, including glucose and lipid metabolism, the inflammatory response, and cell proliferation. PPARs consist of different isoforms (α, β/δ and γ). Lipids provide a range of PPAR ligands, such as fatty acids, eicosanoids, and glucosylceramide (GluCer).

PPARα is highly expressed in the liver and plays a key role in regulating the first step of sphingolipid biosynthesis via SPT.^557,558^ Additionally, GluCer induced by mTORC2 can activate the PPARα pathway to support liver regeneration by promoting facilitates fatty acids oxidation.^559^ Recent studies show that PPARα stimulation leads to hepatocyte hypertrophy in the pericentral region and increased proliferation in the periportal region, promoting liver repair in a YAP/TEAD-dependent manner after PHx.^302,560^ Interestingly, these spatial differences in proliferative capacity are independent of conventional PPARα-mediated fatty acid metabolism.^302^ PPARα activation also regulates FGF21 synthesis, which inhibits oxidative stress and accelerates hepatocyte regeneration in damaged zebrafish livers.^561^ These findings highlight potential therapeutic targets for liver repair and regeneration.

Eicosanoids and long-chain fatty acids are the primary endogenous ligands for PPARβ/δ.^558^ Activation of PPARβ/δ signaling targets both AKT and E2F pathways, influencing liver regeneration by regulating glycolysis, fatty acid synthesis, and cell proliferation following injury.^562^ Additionally, PPAR-δ activation partially mitigates ethanol-induced impairments in liver regeneration by restoring the Wnt signaling pathway.^563^

PPARγ, activated by various arachidonic acid metabolites, is expressed at relatively low levels in the liver.^558^ PPARγ has been shown to delay liver mass restoration in the later stages of liver regeneration after PHx.^564^ Studies indicate that hepatic PPARγ expression contributes to steatosis by promoting triglyceride synthesis and impairing glucose tolerance through monoacylglycerol O-acyltransferase 1 (MGAT1).^565^ Liver-specific *Pparγ*-deficient mice exhibit an enhanced capacity for liver regeneration, mediated by the HGF/c-Met/ERK1/2 signaling pathways.^566,567^

### Amino acid

**Fig. 7:**
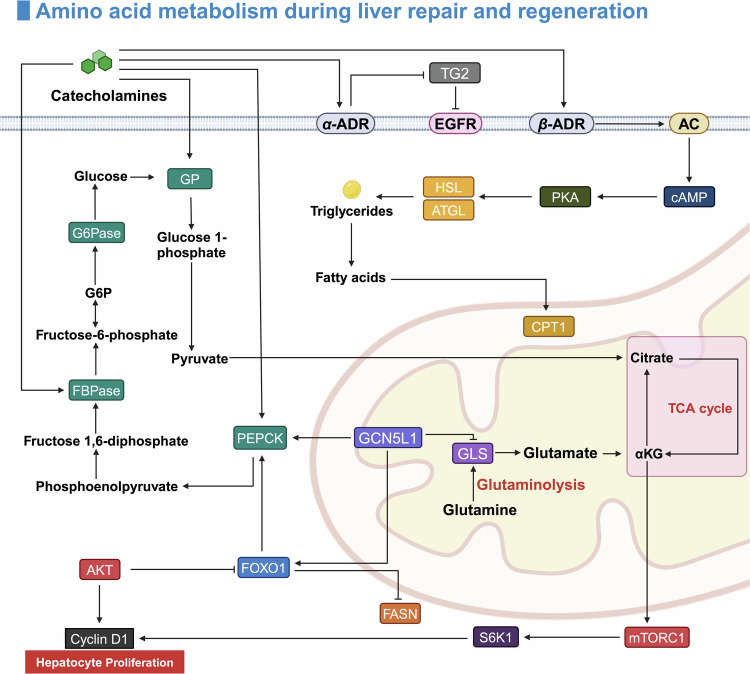
Amino acid metabolism during liver repair and regeneration. Amino acid metabolism involves multiple intracellular and extracellular pathways in different cell subsets. The arrows indicate upregulation or activation, and the T-arrows indicate downregulation or inhibition. TG2 transglutaminase 2, ADR adrenergic receptor, EGFR epidermal growth factor receptor, cAMP cyclic adenosine monophosphate, PKA protein kinase A, AC adenylate cyclase, HSL hormone-sensitive lipase, ATGL adipose triglyceride lipase, CPT1 carnitine palmitoyltransferase 1, TCA tricarboxylic acid, αKG α-ketoglutarate, GLS glutaminase, mTORC1 mammalian target of rapamycin complex 1, S6K1 S6 kinase 1, GCN5L1 general control of amino acid synthesis 5 like 1, FASN fatty acid synthase, FOXO1 forkhead box protein O1, AKT protein kinase B, PEPCK phosphoenolpyruvate carboxykinase, FBPase fructose bisphosphatase GP glycogen phosphorylase, G6P glucose 6-phosphate, G6Pase glucose 6-phosphatase. The figure was generated with BioRender (https://biorender.com)

Amino acid metabolism is crucial for multiple vital functions, including supporting protein synthesis, providing energy, supplying metabolic intermediates, participating in signal transduction, and regulating gene expression. These roles collectively contribute to the complex process of liver repair and recovery, underscoring the importance of amino acids in maintaining hepatic health. During liver regeneration, the demand for amino acids and proteins rises to support new cell synthesis and tissue repair. In this section, we discuss key metabolites and metabolic enzymes involved in liver repair and regeneration **(**Fig. 7**)**.

GSH, an intracellular tripeptide, is involved in several critical functions, including cellular proliferation. During liver regeneration, GSH biosynthesis increases and precedes DNA synthesis.^568^ Reduced GSH levels are associated with delayed hepatocyte proliferation.^569^ Glutamine, synthesized from glutamate by glutamine synthetase, supports GSH production and plays a key role in cellular metabolism. Notably, glutamine can be converted to α-ketoglutarate (αKG), a key intermediate in TCA cycle, through a process called glutamine anaplerosis. This pathway is crucial for supplying energy and biosynthetic precursors during proliferation.^570,571^ Enhanced glutaminolysis, a characteristic of metabolic reprogramming in cancer cells, further promotes rapid cell growth by providing both energy and essential signaling molecules.^572–574^

The general control of amino acid synthesis 5 like 1 (GCN5L1) protein has been shown to modulate mitochondrial protein acetylation. In hepatocyte-specific GCN5L1 KO mice, increased glutamine uptake, along with elevated glutaminase activity via enhanced glutaminase (GLS), synergistically promotes hepatic glutaminolysis. This metabolic reprogramming activates the mTORC1 pathway, partly via elevated αKG levels, ultimately boosting liver regeneration in response to injury.^575–577^ Notably, the inhibition of mTOR signaling significantly delays S phase entry and liver regeneration in an S6K1-dependent manner.^370,578,579^ GCN5L1 ablation also reduces hepatic gluconeogenesis. Mechanistically, GCN5L1 inhibits ERK activation by reducing mitochondrial ROS production, leading to increased FOXO1 levels, which induce the expression of the gluconeogenic enzyme PEPCK and glucose 6-phosphatase (G6Pase).^575^ Furthermore, due to the heterogeneous distribution of hepatocytes, periportal hepatocytes, which are rich in GLS, play a predominant role in regeneration during CCl_4_-induced injury.^580,581^

Catecholamines, synthesized from the amino acid precursors phenylalanine and tyrosine, are a class of neurological substances. Catecholamines can be synthesized by specific activated HSCs.^582–584^ Catecholamines can induce glycogenolysis by activating glycogen phosphorylase (GP) and enhance gluconeogenesis by activating fructose-1,6-bisphosphatase (FBPase) to increase fructose-6-phosphate levels and PEPCK to promote the synthesis of phosphoenolpyruvate.^182^

Catecholamines contribute to triglyceride breakdown into free fatty acids to meet energy demands via a β2-adrenergic receptor (β2-ADR)/cAMP/PKA-dependent mechanism by activating hormone-sensitive lipase (HSL) and adipose triglyceride lipase (ATGL), while also upregulating CPT to enhance fatty acid β-oxidation.^182,585–588^ Beyond their role in energy metabolism, catecholamines also influence liver regeneration. They enhance the effect of EGF on DNA synthesis and prevent transglutaminase 2 (TG2)-mediated transamidation of the EGFR in hepatocytes by binding to α1-adrenergic receptors (α1-ADR)^182,589,590^ Additionally, other research suggests that catecholamines also support the proliferation of cholangiocytes and LPCs.^589,590^

### TCA cycle

The TCA cycle is central to mitochondrial metabolism. In M2 macrophages, the TCA cycle remains intact and well-regulated, while in M1 macrophages, it becomes dysregulated. The downstream metabolism of citrate and succinate is disrupted, leading to the accumulation and escape of these metabolites from the mitochondria.^591,592^ Excess succinate stabilizes HIF-1α, driving the expression of glycolytic genes and reinforcing M1 polarization.^591^

Macrophage-mediated inflammatory responses are essential for liver repair and regeneration. Itaconate, a TCA cycle-derived metabolite produced by macrophages, plays a key role in dampening inflammation and reducing oxidative stress.^593,594^ M2-polarized macrophages show increased production of itaconate, which supports their anti-inflammatory function.^595,596^ Recent studies have demonstrated that itaconate derivative 4-octyl itaconate (OI) also inhibits glycolysis and exerts anti-inflammatory effects by targeting glyceraldehyde 3-phosphate dehydrogenase (GAPDH).^597,598^

In addition to its role in macrophages, itaconate plays a pivotal role in Th17/Treg cell differentiation through metabolic and epigenetic reprogramming. It inhibits both glycolysis and oxidative phosphorylation in these cells, influencing their function.^599^ The balance between Th17 and Treg cells is critical for regulating inflammatory processes and shaping the immune microenvironment, which may synergistically promote liver regeneration.^405,600–602^ These findings underscore the importance of itaconate in immune modulation and metabolic reprogramming, highlighting its potential in liver repair.

### ATP metabolism

The ATP content decreases rapidly by 25% within 15 seconds after PHx.^603^ Further studies have shown that ATP not only acts as an energy source to support regenerative repair but also functions as a purinergic signal, promoting liver regeneration in response to injury.^604^

Liver repair is an energy-intensive process, accompanied by an increased demand for ATP.^451,605,606^ Enhancing bioenergetics can influence cellular metabolism, which is involved in initiating, executing, or terminating the repair process.^607^ ATP activates JNK signaling through paracrine stimulation of P2Y2 receptors, promoting cell cycle progression by driving the G0 to G1 transition in hepatocytes via the activation of Cyclin D1 during liver regeneration.^603,608,609^

Mitochondrial respiratory dysfunction, caused by various factors, impacts ATP production and impairs liver repair.^610–612^ Inhibiting complex I activity and supercomplex formation through depletion of methylation-controlled J (MCJ) protein enhances mitochondrial function and boosts ATP synthesis in the remnant liver. Silencing MCJ can reprogram glycolipid metabolism, improve insulin resistance, and alleviate hepatic steatosis, even overcoming the regenerative limitations in aged livers.^607^ Additionally, the absence of MCJ reduces ROS production, amplifying antioxidant defenses.^613^ This is consistent with previous findings that overactive mitochondrial activity often leads to excessive ROS generation, which exacerbates liver damage.^614–616^

Extracellular ATP also exerts significant influence by regulating liver mass recovery and function through intercellular communication.^603,617^ Elevated extracellular ATP levels facilitate the activation of KCs, leading to the secretion of TNF, IL-6, and EGF, which drive the G1/S transition and promote regenerative progression following PHx.^607^ Upon LPS stimulation, activated macrophages undergo metabolic reprogramming, shifting from oxidative phosphorylation to glycolysis for ATP production, accompanied by the accumulation and oxidation of succinate.^618^ This mitochondrial remodeling supports a proinflammatory state via ROS signaling.^619,620^

Beyond macrophage regulation, extracellular ATP also influences other non-parenchymal cells involved in liver regeneration. Notably, ATP activates P2 receptors on NK cells, inducing IL-22 secretion, which is critical for efficient liver regeneration.^156,164^ Additionally, ATP stimulates liver sinusoidal endothelial cells (LSECs) to express HGF and IL-6 in response to hepatocyte damage, promoting regenerative processes.^617^ However, prolonged exposure to extracellular ATP can lead to LSEC apoptosis and impair angiogenesis through the CD39 pathway, potentially disrupting the repair process.^617^

## Clinical to molecular insights: integrated analysis of repair and regeneration in pathological conditions

**Fig. 8:**
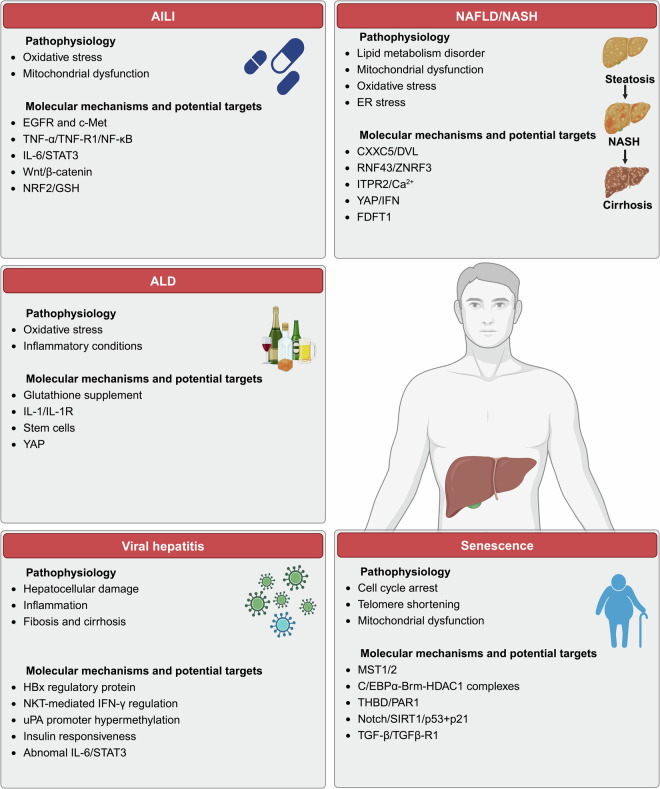
Overview of the molecular mechanisms affecting liver repair and regeneration under pathological conditions. This illustration shows the major pathophysiological mechanisms of various liver diseases and identifies molecular and signaling pathway targets with therapeutic potential. AILI acetaminophen-induced acute liver injury, NAFLD nonalcoholic fatty liver disease, NASH nonalcoholic steatohepatitis, ALD alcohol-related liver disease. The figure was generated with BioRender (https://biorender.com)

Liver disease contributes significantly to global morbidity and mortality, accounting for over two million deaths annually.^621^ The liver’s ability to initiate repair and regeneration in response to both acute and chronic injuries is crucial for maintaining its function and overall health. Acute liver injury from PHx or drug toxicity can activate the liver’s self-repair mechanisms. Chronic conditions such as nonalcoholic fatty liver disease (NAFLD), NASH, alcohol-related liver disease (ALD), and viral hepatitis significantly impair the liver’s regenerative capacity, heightening the risk of cirrhosis and HCC. Additionally, aging reduces the liver’s ability to repair and regenerate, leading to delayed recovery after injury. Metabolic comorbidities, such as diabetes and obesity, further exacerbate liver disease progression and complicate its management.^621^ Investigating alterations in molecular mechanisms related to liver disease and damage repair offers valuable insights into the complexity of these conditions and their treatment **(**Fig. 8**)**.

### Drug-induced liver injury

#### Clinical observations

APAP overdose is the leading cause of drug-induced liver injury and a major contributor to acute liver failure (ALF) and death worldwide. Liver repair and regeneration are crucial in determining the prognosis of patients with AILI. Timely and effective liver regeneration promotes injury resolution, while insufficient or delayed regeneration can lead to an inability to compensate for damage, worsening the condition.^622,623^ Oxidative stress and mitochondrial dysfunction are key cellular events in AILI.^614^ Pathologically, hepatocytes in Zone 3 (around the central vein of the hepatic lobule) are initially affected, followed by necrosis. In severe cases, submassive or massive necrosis may occur. Inflammatory cell infiltration, mitochondrial damage, and apoptosis are also observed.^624^ Diagnosis is facilitated by a clear history of APAP exposure, with liver function tests and blood APAP levels providing additional diagnostic support. Treatment involves discontinuing the suspected drug, administering the detoxifying agent N-acetylcysteine (NAC), and providing prompt systemic supportive care.^614^

#### Molecular mechanisms in liver recovery

Exploring the mechanisms of liver repair and regeneration after APAP-induced hepatotoxicity can potentially improve survival and recovery. Substantial evidence suggests that growth factors and cytokines play important roles in liver repair following AILI. Among these, EGF and HGF are the most extensively studied growth factors. The expression levels of these growth factor-associated receptors, EGFR and c-Met, increase rapidly in mice with moderate APAP administration.^625^ Under moderate doses of APAP, EGFR and c-Met activation promotes tissue repair through protective effects. However, at high doses of APAP, despite similar activation, the liver sustains damage, and recovery is impaired. This contrasting response is likely due to the suppressive signals generated at high doses, which counteract the regenerative effects mediated by EGFR and c-Met, overwhelming their beneficial impact.^626^

Cytokines are also involved in AILI-induced liver repair and regeneration. Increases in the expression levels of TNF-α and downstream NF-κB are positively correlated with the expression of Cyclin-D1 after APAP treatment.^622,627^ TNF-R1 deficiency decreases proliferative signaling after APAP overdose.^628^ Interestingly, low concentrations of TNF-α can induce hepatocyte proliferation by activating YAP, whereas high concentrations trigger cell death by inducing phosphorylation and inactivation of YAP following APAP treatment.^311^ A lack of IL-6/STAT3 signaling activation similarly impairs liver regeneration after AILI.^629,630^ Moreover, phospho-STAT3 expression is delayed in TNF-R1 KO mice.^628^ These findings reveal potential crosstalk between IL-6/STAT3 and TNF-α/ TNF-R1/NF-κB in AILI.^628^ Besides, the inhibitory factor TGF-β also contributes to repair during AILI. Bone marrow-specific TGF-β1 KO or the use of TGFβ-R1 inhibitors significantly improves liver regeneration after AILI.^631^

Wnt signaling is also involved in AILI, with a dose-dependent effect on the Wnt/β-catenin pathway observed following APAP administration.^553,632^ A moderately toxic dose of APAP enhances liver regeneration by promoting β-catenin nuclear translocation, while a severe overdose suppresses β-catenin binding to the *Ccnd1* promoter.^626^

Oxidative stress plays a crucial role in APAP poisoning. The metabolic enzyme CYP2E1 metabolizes APAP into the toxic intermediate N-acetyl-p-benzoquinone imine (NAPQI), which contributes to toxicity during APAP overdose.^633,634^ The proinflammatory molecule OPN mitigates APAP-induced hepatotoxicity by inhibiting CYP2E1 expression.^635^ When NAPQI production exceeds the detoxification capacity of GSH, it binds to intracellular proteins and nucleic acids, leading to mitochondrial oxidative stress.^614,636^ NRF2, a key regulator of antioxidant response, is likely activated by Kelch-like ECH-associated protein 1 (KEAP1) in response to NAPQI.^614,637,638^ NRF2 promotes GSH synthesis and induces the transcription of various antioxidant enzymes.^614,638,639^ Research also shows that HNF4α interacts with NRF2 to replenish GSH, while c-Myc suppresses this process, impacting liver recovery and repair following AILI.^640^

Non-parenchymal cells are integral to AILI. In patients with APAP-induced ALF, severe dysfunction of the hemostatic system is often observed.^641,642^ This dysfunction arises primarily from the accumulation of platelet-VWF aggregates following APAP exposure, which impairs liver repair rather than exacerbating the initial toxicity.^643^

Macrophages, through their plasticity, play a dual role in AILI by modulating the balance between inflammation and repair. Infiltrating macrophages not only amplify inflammation by recruiting CD11b^+^Gr-1^+^ myeloid cells but also contribute to tissue repair by clearing dead cells and promoting neutrophil apoptosis.^119,644^ However, several factors, including the chemokine CCL5 and the metabolite TMAO, can delay liver repair by affecting the activation of alternatively activated macrophages and prolonging the inflammatory response.^119,536^

While APAP-induced liver injury has been extensively studied, investigations into other drugs that cause hepatotoxicity are relatively limited, with CCl4 being one of the primary agents used in such research. CCl_4_ induces liver injury through its metabolism by cytochrome P450 enzymes, which generates trichloromethyl free radicals, causing severe hepatocyte damage through lipid peroxidation and cellular dysfunction.^645–647^ This leads to an inflammatory response, characterized by increased reactive oxygen species and cytokine activity, exacerbating liver damage.^648,649^ In response, the liver initiates a regenerative process that starts with an inflammatory phase to promote hepatocyte survival and proliferation, followed by a proliferative phase where liver cells replicate to restore tissue integrity.^649^ However, chronic CCl_4_ exposure can result in fibrosis due to ongoing collagen deposition by activated HSCs, potentially leading to cirrhosis.^648,650–652^

Liver regeneration following exposure to hepatotoxic agents such as chloroform, trichloroethylene, thioacetamide, and allyl alcohol, which generate reactive metabolites through metabolic activation, similarly induces hepatocyte necrosis.^648,649^ This process involves complex cellular signaling that includes a range of chemokines, cytokines, and growth factors.^623^ The mechanisms underlying these models remain incompletely understood. Continued investigation is essential to fully comprehend liver regeneration and enhance therapeutic strategies for drug-induced liver injuries.

### NAFLD/NASH

#### Clinical observations

NAFLD affect over one-quarter of the global population.^621^ Obesity, metabolic syndrome, diet, lifestyle, genetics, and environmental factors contribute to their development.^621,653^ Diagnosis typically involves a combination of clinical evaluations, laboratory tests, imaging studies, and occasionally liver biopsies. While ultrasound is commonly used as an initial noninvasive method to detect hepatic fat, biopsy remains the gold standard for diagnosis.^654^ The most widely accepted mechanisms underlying the pathogenesis of NAFLD include lipid metabolism disorders, mitochondrial dysfunction, oxidative stress, and ER stress.^655^ NAFLD begins with lipid accumulation in hepatocytes. As steatosis advances, inflammation triggers hepatocyte apoptosis, necrosis, and fibrosis, progressing to NASH.^656^ While TRAS play a key role in early liver regeneration, the worsening of steatosis and inflammation ultimately compromises regenerative potential, leading to irreversible damage, potentially cirrhosis or HCC.^657,658^ Consistent with this, obesity is also associated with delayed regenerative capacity compared with non-obese individuals.^659^ Severe hepatic steatosis increases the risk of primary graft nonfunction and compromised liver transplantation outcomes.^660^ Therefore, maintaining lipid metabolic balance is essential for effective liver regeneration.

Although the FDA has approved Resmetirom as a new treatment option,^661^ lifestyle interventions targeting NAFLD/NASH risk factors, such as weight loss through a healthy diet and physical activity, are expected to remain central to improving patient outcomes.^653,656^ Alongside lifestyle modifications, numerous modulators targeting key enzymes involved in fatty acid metabolism, including ACC, FAS, SCD1, diacylglycerol acyltransferase (DGAT), and PFK1, are in clinical development, with combination drug strategies being actively explored.^662^ Given the strong link between lipid metabolism and the gut microbiota, innovative microbiome-mediated therapies, such as fecal microbiota transplantation, engineered bacteria, and bacteriophages, are also emerging as promising approaches to complement existing treatments.^663^

While recent discussions have increasingly advocated for the adoption of MAFLD (metabolic dysfunction-associated fatty liver disease) to emphasize the role of metabolic disorders in disease pathogenesis, much of the existing literature has not incorporated this perspective. Accordingly, this review continues to use the terms NAFLD/NASH, in line with the terminology widely used in prior studies, while acknowledging the ongoing shift in the field towards a more metabolically focused classification.

#### Molecular mechanisms in liver recovery

Wnt/β-catenin signaling, which is implicated in various metabolic disorders, is suppressed in the livers of patients with NASH.^664^ Studies have shown that CXXC5 KO rescues diet-induced metabolic dysfunction and prevents NASH development. The small molecule KY19334, which inhibits the CXXC5/DVL interaction, reactivates Wnt/β-catenin signaling, ameliorating liver tissue injury by suppressing pathological phenotypes and enhancing regenerative processes in NASH.^266^ Additionally, liver-specific deletion of RNF43/ZNRF3 leads to increased levels of unsaturated lipids and the development of steatohepatitis, while also causing uncontrolled regenerative capacity following chronic damage, thereby increasing the risk of carcinogenesis.^266^ Thus, identifying and managing individuals at high risk for genetic mutations in the Wnt signaling pathway is critical.

Ca^2+^ is a ubiquitous second messenger involved in both physiological and pathological processes including hepatocyte proliferation.^665^ The subcellular regulation of Ca^2+^ relies in part on the type II inositol 1,4,5-trisphosphate receptor (ITPR2), located on the ER membrane. When activated, ITPR2 releases Ca^2+^ from the ER into the cytoplasm. In NAFLD, however, ITPR2 expression is reduced due to elevated c-Jun levels, impairing liver regeneration.^666^

ALR, a key protein for promoting liver regeneration, plays a vital role in maintaining lipid homeostasis and mitochondrial function.^179^ In NASH patients, ALR levels are significantly reduced, and this deficiency may contribute to disease progression.^180^ Effectors of inflammation in NASH negatively regulate ALR expression, though the precise mechanisms remain unclear.^667^ This establishes a potential link between ALR-mediated liver regeneration and NASH. Notably, ALR degradation via ubiquitination exacerbates mitochondrial fission, promoting the progression from NASH to HCC.^668^

Fibrosis is a hallmark of NASH and a significant risk factor for cirrhosis and liver failure.^669^ Chronic or severe liver damage often results in maladaptive repair, leading to fibrosis and disrupting the delicate balance between regeneration and scarring. This balance is influenced by complex cellular interactions.^670,671^ In NASH models, myeloid-specific YAP deficiency has been shown to suppress fibrogenesis and promote hepatocyte proliferation through the activation of type I IFN signaling.^672^ Additionally, endothelial cells play a key role in supporting liver repair via paracrine and angiocrine signaling.^670,673^ Aberrant epigenetic modifications within endothelial cells may further impair liver regeneration,^674^ highlighting potential therapeutic targets to restore regenerative capacity in NASH.

Alleviating disease progression and improving liver histology are critical goals in treating NAFLD/NASH. The recent FDA approval of Resmetirom marks a significant breakthrough. This drug selectively targets thyroid hormone receptor-β in the liver and has demonstrated efficacy in clinical trials for treating patients with noncirrhotic NASH and moderate to advanced fibrosis.^661,675^ Researchers continue to explore additional treatment modalities. Bavachinin, a natural compound from Fructus Psoraleae, has been shown to reduce lipid accumulation in NAFLD by promoting fatty acid oxidation and inhibiting the cholesterol synthesis enzyme Farnesyl-diphosphate farnesyltransferase 1 (FDFT1), while also enhancing cell proliferation through its facilitation of the interaction between PCNA and DNA polymerase delta.^676,677^

Given the complex nature of NAFLD/NASH and its diverse pathological features, including insulin resistance, steatosis, inflammation, and fibrosis, achieving effective treatment remains a significant challenge. Considering the therapeutic limitations of targeting individual phenotypes, future research should prioritize a more comprehensive approach to the disease’s overall pathogenic processes.

### ALD

#### Clinical observations

ALD is one of the leading causes of mortality worldwide.^621^ The spectrum of ALD includes liver steatosis, steatohepatitis, hepatitis, cirrhosis, and HCC, with disease progression occurring through these stages.^678^ Acute alcoholic hepatitis (AH) can lead to acute-on-chronic liver failure (ACLF) and carries a high risk of short-term mortality.^679^ Persistent excessive alcohol intake causes both direct hepatocyte damage and gut-liver axis disruption, leading to indirect injury.^680–682^ Diagnosis of alcoholic hepatitis is based on a history of alcohol consumption, clinical symptoms, and supporting lab and imaging studies.^683,684^

Abstinence from alcohol is the most crucial step in treating ALD, as it can prevent further damage and potentially reverse some effects, especially in early stages.^683,685^ Nutritional support and medications are used to manage complications, with corticosteroids currently the only approved treatment for severe AH.^686,687^ Understanding the mechanisms of liver injury in ALD is vital for developing future therapeutic strategies.^684^

#### Molecular mechanisms in liver recovery

Accumulating evidence indicates that oxidative stress and inflammation are primary drivers of cellular damage in AH.^678,684,688^ Alcohol metabolism leads to increased lipid accumulation, depletion of the antioxidant glutathione, and mitochondrial dysfunction through ROS-induced oxidative stress.^689–691^ Despite ongoing preclinical studies and clinical trials, effective antioxidants that improve the long-term prognosis of AH patients remain lacking.^678^

The process of liver regeneration during the severe inflammatory response in AH involves complex cellular crosstalk mediated by inflammatory cytokines and chemokines. Elevated IL-1 levels in AH patients are closely linked to inflammation. Preclinical data suggest that blocking the IL-1 receptor reduces inflammation and promotes liver regeneration.^692,693^ Several randomized trials have also demonstrated that G-CSF mobilizes hematopoietic stem cells, leading to improved patient survival.^694–696^ Additionally, YAP dysregulation in AH may impair liver repair and regeneration by regulating metabolic enzymes, making it a promising therapeutic target.^697,698^

### Viral hepatitis

#### Clinical observations

Viral hepatitis is a major risk factor for chronic liver disease and HCC, contributing to significant mortality worldwide, with hepatitis B and C accounting for the greatest global burden.^621^ It typically presents as hepatocellular damage, inflammation, and cholestasis, which can lead to jaundice. As the disease progresses, chronic infection often leads to fibrosis and cirrhosis, marked by scar tissue formation and diminished liver function.^699,700^ In advanced stages, structural changes such as regenerative nodules, lobular disruption, and portal hypertension, along with related complications, are common.^701^ Diagnosis usually involves liver function tests, serological markers, and viral load quantification.^702^

Antiviral therapy remains the primary treatment, depending on the virus type and disease stage.^703,704^ Additionally, symptomatic and supportive care, along with complication management, are important. For advanced or severe cases, liver transplantation may be required.^705–707^

#### Molecular mechanisms in liver recovery

Among the various hepatitis viruses, HBV has been extensively studied for its impact on liver regeneration. The HBV X protein (HBx), a multifunctional regulatory protein, plays a critical role in the pathogenesis of HBV infection by modulating transcription, signal transduction, cell cycle progression, and apoptosis.^708^ Research has shown that HBx impairs liver regeneration through multiple mechanisms. In HBx-transgenic mice, liver regeneration capacity is reduced following PHx, as HBx disrupts the cell cycle by inhibiting the G1/S phase transition and altering the expression of key regulators such as p21 and PCNA.^709–711^ This disruption has significant implications for liver regeneration and disease progression in HBV-infected patients. Additionally, activated NKT cells further inhibit liver regeneration in HBV-transgenic mice through IFN-γ-mediated pathways.^138^ Epigenetic alterations, such as the hypermethylation of the uPA promoter, have also been observed, leading to disruptions in HGF-mediated liver regeneration.^712^

Alterations in signaling pathways are prominent in HBV models. HBV activates NRF2 to increase insulin receptor expression but impairs its transport to the plasma membrane via α-taxilin, reducing insulin sensitivity and suppressing regeneration.^713,714^ Paradoxically, NRF2 also prevents oxidative stress and apoptosis in HBV-infected cells by inducing ALR expression, thereby supporting viral replication.^715,716^ This protective mechanism, which promotes cell proliferation and immune evasion, is similarly observed in HCV infection and may contribute to cancer susceptibility.^717,718^ Interestingly, in HBx-transgenic mice, increased IL-6 and STAT3 phosphorylation accompany inhibited cell cycle progression following PHx.^719^ Additionally, in vitro studies have shown that HBx blocks the transcriptional activity of p53 via long noncoding RNA, promoting hepatocyte proliferation.^720^ These abnormal proliferation-related signals may also be associated with accelerated tumorigenesis.^721^

### Senescence

#### Clinical observations

As the population ages, senescence has become an increasingly important factor in liver regeneration. Aging disrupts this process through cell cycle arrest mediated by p53 and p21, which halt hepatocyte proliferation, particularly in chronic injury or disease.^376,722–724^ Telomere shortening, another hallmark of aging, contributes to the senescent phenotype. Telomere shortening reflects the cumulative cellular damage over time, exacerbating fibrosis and reducing liver function during chronic disease progression.^725–727^

Senescence-induced physiological and metabolic alterations significantly impact liver health and its ability to regenerate.^723^ Mitochondrial dysfunction in senescent cells impair oxidative phosphorylation, increase ROS production, and disrupt fatty acid oxidation, leading to lipid accumulation and promoting steatosis and diseases such as NASH.^723,728–732^

Understanding how senescence influences liver repair and regeneration is crucial for developing targeted strategies to improve quality of life and extend longevity in the elderly.

#### Molecular mechanisms in liver recovery

Aging is marked by a progressive decline in tissue and organ functionality and integrity.^733^ Studies have shown that the proportion of hepatocytes proliferating after hepatectomy in aged mice is significantly lower than in young mice, highlighting the impact of aging on liver regeneration.^734,735^ Senescent cells are characterized by cell cycle arrest, increased autophagy, and heightened apoptosis, along with markers such as p21 and the senescence-associated secretory phenotype (SASP).^723,736,737^

The aged liver exhibits a reduced regenerative capacity.^738,739^ During cellular senescence, shifts in interactions between key proteins alter the normal functions of associated signaling pathways. For example, studies have shown that suppressing MST1/2, a key enzyme in the Hippo signaling pathway, can rescue impaired liver regeneration in senescent mice.^282^ This underscores the potential for targeting specific pathways to enhance liver regeneration in senescent liver.

Another key factor contributing to the reduced regenerative ability of the aged liver is epigenetic alterations,^740^ particularly involving CCAAT/enhancer binding protein alpha (C/EBPα). In aging mice, C/EBPα forms complexes with the epigenetic regulators Brm and HDAC1, which modify chromatin structure by deacetylating histones and inducing trimethylation at H3K9.^738,741–743^ This epigenetic suppression directly inhibits the expression of key regenerative genes, such as *Foxm1b* and *c-myc*, both essential for liver regeneration.^744–746^ Interestingly, the epigenetic changes can be reversed during regeneration,^747^ highlighting the dynamic interplay between cellular proliferation and age-related epigenetic modifications.

Senescence creates a more complex and often less favorable microenvironment.^739^ Recent studies have highlighted the growing impact of senescent hepatocyte accumulation as fibrosis progresses in NASH. These senescent cells activate the thrombomodulin–protease-activated receptor-1 (THBD–PAR1) signaling pathway, which induces Hedgehog expression, perpetuating maladaptive repair and fibrosis through non-parenchymal cells.^748^ Inhibition of this pathway using the PAR1 antagonist vorapaxar has been shown to reduce the burden of senescent cells, offering a potential therapeutic strategy.^748^ Additionally, altered vascular shear stress contributes to LSEC senescence, disrupting sinusoidal remodeling and delaying liver regeneration. Mechanistically, reduced shear stress after PHx exacerbates LSEC senescence through the activation of Notch signaling, which inhibit SIRT1 expression. This leads to the activation of p53-, p21- and p16-dependent canonical senescence pathways, further impeding liver regeneration.^749–752^ Moreover, macrophages significantly influence liver aging, particularly through TGF-β signaling. In AILI-induced hepatocellular senescence, macrophage-derived TGF-β drives the process. Inhibition of this pathway with the TGFβ-R1 inhibitor AZ12601011 suppresses senescence, reduces liver injury, and enhances regeneration.^631^

### Comparative analysis of liver repair and regeneration mechanisms across pathological conditions

Liver repair and regeneration are essential for recovery from various injuries, involving complex cellular and molecular interactions. To comprehensively understand liver regeneration across various disease contexts, it is crucial to examine the two most studied models: AILI and PHx. AILI presents a model where regeneration is hindered by chemical toxicity, inducing oxidative stress and mitochondrial damage, which significantly challenge the liver’s regenerative capabilities.^119,614^ In contrast, the PHx model represents a simpler scenario of tissue loss.^753^ Regeneration is initiated by surgical removal of liver tissue, primarily driven by growth factors such as HGF and EGF.^1^ This process is less complicated by oxidative damage, allowing a more direct path to tissue recovery.

Hemodynamic changes, including increased portal vein pressure and altered hepatic blood flow, trigger mechanical and biochemical signals critical for initiating regeneration following PHx.^1^ These changes involve shear stress on liver sinusoidal endothelial cells and arteriolar sphincter adjustments to maintain microcirculatory balance, creating a favorable environment for liver regeneration.^244,754,755^

The pattern of hepatocyte proliferation also differs between these models. In the PHx model, the entire remnant liver undergoes regeneration, whereas in toxic injuries like APAP, proliferation is primarily localized to the cells surrounding necrotic zones.^100,119,753,756^ This results in an unsynchronized cell cycle during regeneration following AILI.^753^ Recent findings from human single-cell studies reveal that ANXA2^+^ migratory hepatocytes emerge in AILI, initially migrating to necrotic sites for wound closure, followed by proliferation, thereby integrating migration and proliferation in a unique regenerative mechanism.^756^

The differences observed in AILI and PHx exemplify how specific liver injuries uniquely shape regenerative pathways. Building on these two models, liver regeneration across a broader range of disease contexts also displays both shared mechanisms and distinct features shaped by specific pathophysiological and molecular factors.

Liver repair and regeneration exhibit several commonalities across different conditions, such as the activation of inflammatory and growth factor signaling pathways, including TNF-α, IL-6, HGF, and EGF, which play crucial roles in hepatocyte proliferation and tissue repair.^311,712,757–759^ Cellular crosstalk between parenchymal and non-parenchymal cells, facilitated by cytokines, chemokines, and growth factors, is essential for orchestrating liver repair across various pathological contexts.^86,604^ The consistent involvement of key pathways like Wnt/β-catenin further underscores the fundamental mechanisms that drive liver regeneration.^266,626,760^

Each liver condition presents unique challenges to its regenerative capabilities. In ALD, persistent alcohol consumption exacerbates liver damage, complicating regeneration differently compared to conditions such as NAFLD, where metabolic factors play more significant roles.^761^ Moreover, NAFLD and NASH are frequently linked to metabolic disorders like obesity and diabetes, which further complicate the liver’s ability to repair and regenerate.^762^ Notably, acute injuries generally trigger rapid repair mechanisms to restore liver function and often retain better regenerative potential as long as the liver structure remains intact. In contrast, chronic conditions involve complex interactions of fibrosis, inflammation, and progressive cellular damage, fundamentally impairing regeneration due to disrupted tissue architecture.^614,763^

Understanding these differences highlights the adaptability of liver regeneration and informs future personalized therapies. Aligning targeted treatments with specific regenerative mechanisms holds promise for significantly improving clinical outcomes.

## Innovations in analytical methods and therapeutic strategies

### Advances in analytic methods

**Fig. 9:**
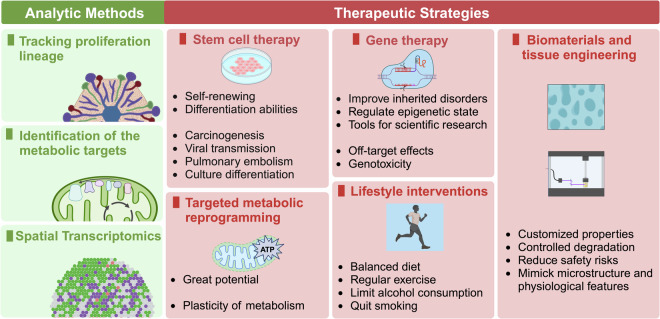
Innovations in analytical methods and therapeutic strategies. This illustration summarizes the characteristics of currently available treatment strategies and potential future treatments and highlights diagnostic approaches that can be used to explore new therapeutic targets. The figure was generated with BioRender (https://biorender.com)

Advancements in detection technologies have greatly enhanced our understanding of liver repair and regeneration. These methods enable researchers to observe liver cells and tissues with unprecedented precision, revealing key regenerative processes in real-time and providing deeper insights into this complex phenomenon. By identifying crucial molecules and signaling pathways involved in injury recovery, these technologies pave the way for new therapeutic strategies (Fig. 9).

#### Tracking proliferation lineages

Identifying the cellular origins and favorable niches for regenerated liver tissue is crucial, particularly in the context of different diseases.^764^ Cell proliferation forms the basis of repair and regeneration, and traditional methods for assessing proliferation can be grouped into three categories: cell proliferation marker staining, nucleotide analog incorporation, and isotope labeling. However, these conventional techniques have significant limitations. Immunostaining provides only a snapshot of cell proliferation without capturing dynamic changes in the regenerative process. Nucleotide analog incorporation allows extended observation but poses cytotoxic risks, while isotope labeling is cumbersome and inconvenient.^765^ Furthermore, none of these methods effectively assess cell type-specific proliferation.

The contribution of different hepatic cells to repair has become a focal point of research. The liver’s basic structural unit, the hepatic lobule, displays significant heterogeneity in metabolic function and gene expression along its axis, from the portal vein to the central vein. Based on these differences, hepatocytes are categorized into three zones: periportal hepatocytes (Zone 1), midlobular hepatocytes (Zone 2), and pericentral venous hepatocytes (Zone 3).^766^ Lineage tracing provides insights into the origins of new liver cells by labeling those expressing specific genes, with the Cre–loxP recombination system being the most commonly used technique.^767,768^ This genetic labeling system ensures stable inheritance and expression of reporter genes, allowing the tracing of proliferation and differentiation of specific cell populations during liver repair and regeneration.^769^ However, the accuracy of this technique largely depends on Cre specificity,^770,771^ and non-specific expression or reduced Cre activity can confound fate-mapping results.^249,772,773^ Continuous tamoxifen treatment, required to maintain Cre activity, also poses potential toxicity concerns.^774^

A recently developed proliferation lineage tracing method, ProTracer, enables continuous, high-resolution in vivo tracking of specific cell proliferation.^46^ ProTracer uses a dual Cre- and Dre-mediated genetic system, providing an unbiased approach for tracing the proliferation of hepatic cell populations.^46^ Research has shown that Zone 2 hepatocytes contribute the most to hepatocyte proliferation for liver homeostasis and injury repair.^46,775^

#### Spatial transcriptomics

Hepatocyte zonation within the liver lobule reflects significant functional variations driven by gradients of oxygen, nutrients, hormones, and Wnt morphogens.^45^ Advances in genome-scale imaging have provided deeper insights into cell profiling and spatial tissue mapping.^776–778^ Halpern et al. used scRNA-seq and single-molecule fluorescence in situ hybridization to analyze molecular expression differences across liver zones, showing that approximately 50% of liver genes exhibit significant zonation.^45,779^ Beyond hepatocytes, other liver cell types—including LSECs, HSCs, and KCs—also display zonated distributions and functions.^780,781^ This zonation enables efficient coordination of metabolism, detoxification, immune response, and tissue repair, essential for liver health.^782,783^

Liver regeneration is a highly coordinated process that relies on complex intracellular and intercellular signaling networks.^784^ Integrating the spatial and temporal dynamics of multicellular interactions during zonal liver regeneration provides a comprehensive understanding of injury repair. Following AILI, mitotic pressure drives midlobular hepatocytes into the pericentral zone, where they replace damaged cells.^781^ This adaptation involves not only localized proliferation and displacement but also significant reprogramming of cellular identity, with transient expression of fetal liver genes such as *Afp*, *Cdh17*, and *Spp1*.^781^ Additionally, hepatocytes near the injury site upregulate Wnt/β-catenin target genes and pericentral metabolic enzymes regulated by Wnt/β-catenin, consistent with the regenerative effects promoted by Wnt2 and Wnt9b in pericentral endothelial cells.^236,784^ This highlights coordinated regional liver regeneration among different cell types. Genetic lineage tracing has shown that 84% of newly formed cells originate from CYP2E1^+^ cells located around necrotic areas in a CCl_4_-induced liver injury model.^785^ These high-resolution spatiotemporal maps provide valuable insights into zone-specific cues that contribute uniquely to the regenerative process.

#### Identification of metabolic targets

The regenerative capacity of the liver depends not only on the proliferation of liver cells but also on a highly coordinated process driven by the cellular metabolic network, which involves unique metabolic traits across heterogeneous cell subgroups that are gaining increasing research attention.^784,786^ Monitoring cellular metabolic adaptation, which involves metabolic flexibility and plasticity, remains a significant challenge in understanding metabolic remodeling during liver repair and regeneration.^386^

Advances in high-throughput and high-resolution technologies have helped address these challenges. Recent developments in multiomics imaging have provided new insights into detailed profiling of individual cells and the spatial mapping of tissues.^776,777,786^ Combining multiomics analyses, including genomics, transcriptomics, proteomics, and metabolomics, offers a systematic view of metabolic reprogramming during liver regeneration, contributing to the identification of new therapeutic targets to enhance regenerative capacity.^387,787–790^

In particular, the integration of data from scRNA-seq and spatial transcriptomics has provided more comprehensive insights into the cellular transcriptome and spatial context.^778^ scRNA-seq effectively reveals the expression patterns of metabolites and metabolic pathways across different cell types, while spatial transcriptomics rapidly captures in situ transcriptomic information for entire tissues.^791^ The combination of spatial and temporal dynamics in multicellular zonal liver regeneration provides a comprehensive understanding of injury repair orchestrated by diverse hepatic cell subtypes.^781,784^

Tracking changes in cellular metabolism during metabolic adaptation is also crucial. The use of stable isotopes in vivo to trace metabolic flux represents an important direction for future research.^792,793^

### Current and future therapeutic strategies

Numerous signaling pathways significantly contribute to liver repair and regeneration, facilitating hepatocyte proliferation and non-parenchymal cell transdifferentiation. Exploring the crosstalk among these pathways and the regulatory mechanisms that govern them is critical for developing therapeutic strategies.

#### Stem cell therapy

Before the advent of cell therapy, liver transplantation was the only effective treatment for end-stage liver disease caused by impaired regenerative capacity. Cell therapy utilizes various cell types to reconstruct or replace damaged tissues, aiming to stimulate repair.^794,795^ Stem cell-based therapies hold promise in reducing the need for liver transplants and improving outcomes for patients with end-stage liver disease. The first type of cell therapy for liver disease involved hepatocyte transplantation. However, issues with hepatocyte acquisition, preservation, and immunological rejection limit its feasibility.^36^ Stem cell transplantation offers a promising alternative for treating advanced liver diseases by harnessing the self-renewal and differentiation abilities of stem cells. Several types of stem cells are used, including mesenchymal stem/stromal cells (MSCs), induced pluripotent stem cells (iPSCs), fetal liver stem cells, and hematopoietic stem/progenitor cells.^794–796^

MSC-based therapies have garnered considerable interest due to their ability to enhance liver regeneration and mitigate liver damage. MSCs can be sourced from various tissues, including the brain, thymus, heart, liver, and lungs, and are easily expanded in culture, making them ideal candidates for transplantation.^797^ Their high plasticity, low immunogenicity, and immunomodulatory properties make MSCs promising for treating liver failure. Transplanted MSCs migrate to injured tissues, stimulating liver regeneration by promoting hepatocyte proliferation, reducing fat accumulation, and facilitating paracrine mechanisms.^797,798^ MSCs also enhance liver glycogen production by suppressing GSK-3β and stimulate cell growth through the GSK-3β/β-catenin pathway.^799^ Additionally, MSC transplantation activates the mTOR pathway and ameliorates mitochondrial damage via IL-10 secretion after PHx, promoting β-oxidation and liver regeneration.^447^ MSC-derived extracellular vesicles (EVs) and exosomes have also been shown to improve liver regeneration in chronic liver disease and aged liver.^800,801^

Despite these benefits, potential risks of MSC treatment must be considered, including carcinogenesis, viral transmission, pulmonary embolism, and differentiation from long-term culture.^802,803^ Future research should focus on enhancing the safety and efficacy of these therapies. Cell-free EVs, which act directly on damaged areas, are being developed as alternatives to address the limitations of cell-based therapies.^801^ Moreover, pretreatment strategies can improve cell resistance to pathological conditions and enhance survival.^803^ The combination of biomaterials and tissue engineering approaches, such as hydrogels and scaffolds, also offers a supportive microenvironment for cell transplantation.^804^

#### Gene therapy

Advances in sequencing technologies and bioinformatics tools have greatly enhanced our understanding of the genetic origins of inherited disorders. The liver, as a central metabolic hub, plays an important role in many genetic metabolic disorders.^805^ Consequently, the liver is a promising target for gene therapy development. Gene editing, gene addition, mRNA therapy, and gene silencing represent powerful tools for studying liver biology and developing new therapeutic strategies.^805,806^

CRISPR-Cas9 is a revolutionary and widely used gene-editing tool, notable for its precision, simplicity, and versatility in modifying genetic sequences. This technology has also been adapted to influence epigenetic states, further expanding its potential in research and therapeutic applications.^807,808^ However, off-target effects and genotoxicity remain significant concerns in the therapeutic use of CRISPR-Cas9. Accuracy- and safety-related risks continue to constrain its clinical application. Future research should focus on improving CRISPR delivery systems and enhancing spatiotemporal control in biological tissues.^807,808^

Despite these challenges, the use of gene editing in xenotransplantation is an important breakthrough, offering new possibilities for overcoming organ shortages. Gene editing technologies, particularly CRISPR-Cas9, allow precise genome modifications in animals, reducing immune rejection and improving organ compatibility.^809,810^

#### Biomaterials and tissue engineering

The process of liver repair and regeneration relies on the ECM and the extracellular niche, which support cell–matrix and cell–cell interactions. Designed to mimic the ECM, synthetic or natural biocompatible materials can guide appropriate cellular responses during injury repair. These biomaterials come in various forms, including polysaccharides, proteins, aliphatic polyesters, nanofibers, and decellularized materials.^329,811^ Compared to natural materials, synthetic materials in liver tissue engineering offer greater batch-to-batch consistency, customizable mechanical properties, controllable degradation rates, and reduced biological safety risks. However, synthetic materials may lack the specific biological signals and microenvironments present in natural materials that are crucial for cell behavior and tissue regeneration.^812^ Therefore, selecting the appropriate material requires balancing these factors based on the specific needs and objectives of the application.

Moreover, the complex microarchitecture of the liver requires compatible physical properties to maintain cellular function. A major challenge in fabricating bioapplicable materials is replicating the intricate hepatic microstructures and physiological characteristics. Several tissue engineering approaches, such as cell sheets, scaffolds, hydrogels, microspheres, 3D bioprinting, microfluidic systems, and liver organoids, have been used to mimic the hepatic environment.^811,813^ Before clinical application, it is critical to evaluate whether implanted grafts can maintain cellular phenotype and function across different liver disease contexts.

#### Targeted metabolic reprogramming

Cellular metabolism plays a crucial role in injury recovery by providing essential energy and acting as signaling molecules.^547,671,814^ Targeting specific metabolic pathways in distinct liver cell types is a promising strategy, informed by insights into metabolic changes during liver repair and regeneration.^43,607,815^ However, the plasticity of cellular metabolism allows cells to adapt to alternative pathways when specific metabolic routes are blocked.^11,62,796^ Moreover, complex crosstalk between extrahepatic and intracellular pathways, as well as the presence of multiple isoforms of key metabolic enzymes, can limit the effectiveness of such treatments.^274^

Despite these challenges, promising preclinical evidence supports the potential of targeting metabolic processes in liver regeneration. Future research should focus on validating these targets and translating them into novel therapeutic approaches, including the development of specific activators and inhibitors.

#### Lifestyle interventions

Lifestyle plays a crucial role in liver health management and influences various liver diseases, including those associated with metabolic syndrome.^653,816–818^ Lifestyle modifications may offer a promising direction for enhancing liver regeneration. A balanced diet is essential for maintaining liver homeostasis and function. In a randomized controlled study, Gupta et al. demonstrated that lifestyle optimization, including a healthy diet and regular exercise, significantly improved liver regeneration in live liver donors and reduced the incidence of early graft dysfunction in recipients.^819^ Limiting alcohol intake and quitting smoking also reduce the risk of liver inflammation and damage.^688,820^ As our understanding of injury recovery mechanisms advances, lifestyle modifications could become a key strategy for enhancing liver regeneration and improving disease treatment.

## Clinical trials and research progress

Although preclinical studies have extensively explored the molecular mechanisms of liver regeneration, the complex functions of the liver and its regeneration process pose significant challenges in terms of safety and complexity for conducting clinical research. Currently, clinical research on liver regeneration has focused primarily on cell and cytokine therapies,^694,821,822^ pharmacological interventions,^823,824^ and improvements in surgical techniques (Table 2).^825,826^ These studies have led to many meaningful explorations.

**Table 2:** Clinical trials regarding liver repair and regeneration

Treatment strategies	Intervention	Location	Trial number	Type of trial	Phase	Enrollment	Status	Study population	Ref.
Behavior	Donor lifestyle optimization	India	NCT04565535	Randomized control pilot study	Not applicable	75	Completed	Live liver donors	^819^
Cell Therapy	Platelet activation	Austria	NCT02113059	Observational	Not applicable	40	Unknown	Partial hepatic resection	
	Bone marrow-derived mesenchymal stem cells	Japan	NCT02327832	Interventional	Phase 1	10	Unknown	Decompensated cirrhosis	
	Autologous peripheral stem cells transplantation	Korea	NCT01108380	Interventional	Not applicable	30	Unknown	Extensive hepatectomy	
	Adipose tissue derived stromal cells	Japan	NCT00913289	Unknown	Phase 1	6	Terminated	Liver cirrhosis	
	Cells intraportal infusion of autologous bone marrow mononuclear	Spain	NCT01745731	Randomized controlled trial	Phase 2	13	Completed	Partial hepatic resection	
	Marrow stem cell	Switzerland	ISRCTN83972743	Single-center, randomized controlled study, not blinded	Not applicable	60	Completed	Decompensated alcoholic liver disease	^821^
Cytokine Therapy	G-CSF+erythropoetin	India	NCT01902511	Randomized double blind study	Not applicable	60	Completed	Decompensated cirrhosis	^822^
	G-CSF	Brazil	ISRCTN16342840	Randomized Controlled Trial	Not applicable	79	Completed	Benign or malignant liver tumors	
	G-CSF	Switzerland	ISRCTN86571875	Randomized controlled pilot trial, not blinded	Not applicable	24	Completed	Alcoholic steatohepatitis	^694^
Drug	Phosphate supplementation	India	NCT04026438	Randomized controlled trial	Phase 4	130	Completed	Live liver donors	
	Pentoxyfilline	Switzerland	NCT00957619	Randomized prospective trial	Phase 4	100	Completed	Partial hepatic resection	^823^
	High-dose insulin	Canada	NCT06126419	Interventional	Not applicable	70	Recruiting	Major hepatic resection	
	Ursodeoxycholic acid	India	NCT06091787	Open label randomized trial	Not applicable	80	Unknown	Right lobe donor hepatectomy	
	Rifaximin	Germany	NCT02555293	Single-center, open-label, randomized controlled trial	Phase 3	96	Terminated	Major hepatic resection	^824^
	Propofol	France	NCT00219856	Prospective, randomized, simple blind study	Phase 3	34	Completed	Partial hepatic resection	
	N-acetylcysteine	Canada	ISRCTN01624686	Single-center, randomized clinical trial, not blinded	Not applicable	460	Completed	Major hepatic resection	
Surgery	Selective reversible PVE	France	NCT02945059	Multicentric prospective study	Not applicable	33	Completed	Major hepatic resection	
	Radiofrequency assisted liver partition with PVL	UK	NCT02216773	Single-center, prospective, randomized controlled trial	Not applicable	57	Completed	Partial hepatic resection	
	Bariatric surgery	Germany	NCT02792634	Observational	Not applicable	200	Unknown	Liver regeneration after bariatric surgery	
	Splenic artery ligation	Greece	NCT05459883	Observational	Not applicable	13	Completed	Major liver resections with evidence of small-for-size syndrome	
	Liver venous deprivation	France	NCT03841305	Multicentric comparative randomized trial	Phase 2	64	Recruiting	Colo-rectal liver metastases	^825^
	PVE with radio frequency ablation	Denmark	NCT04107324	Randomized controlled trial	Not applicable	30	Unknown	Colo-rectal liver metastases	
	PVE with micro-particles plus coils, PVE with n-butyl-cyanoacrylate	Portugal	ISRCTN16062796	Randomized controlled trial	Not applicable	60	Completed	Liver cancer	^826^

Cell and cytokine therapeutic applications for liver regeneration are under active investigation, yet definitive conclusions regarding their efficacy remain elusive. Although these therapeutic strategies hold potential, their effectiveness in different liver disease contexts still requires substantial evidence.^694,821,822^

Pharmacological interventions hold potential for liver regeneration. Although these treatments show promise, definitive evidence of their efficacy has yet to be established, and the potential side effects require careful consideration to ensure their safety and effectiveness in clinical use.^823,824^. In a double-blind, randomized, controlled trial involving 101 patients who underwent major hepatectomy, the perioperative administration of pentoxifylline significantly increased the regenerative capacity of small remnant livers (remnant liver to body weight ≤ 1.2%). This proliferative effect is likely mediated by IL-6.^823^ However, the higher incidence of drug-related adverse events in the pentoxifylline group may limit its application.

Encouragingly, recent clinical studies have shown that HRX215, a novel MKK4 inhibitor, significantly enhances liver regeneration in mouse and pig models after PHx.^827^ Treatment with HRX215 reduces mortality rates following critical 85% hepatectomy and hampers the progression of NASH-related HCC. Moreover, HRX215 has potent anti-steatotic and anti-fibrotic effects. The preliminary human phase I trial has shown favorable pharmacokinetics and safety profiles, indicating the potential of HRX215 to prevent liver failure associated with small-for-size syndrome after extensive liver resection. This advancement could broaden the scope of surgical resections and benefit more patients.^827^

Surgical advancements focus on reducing trauma, enhancing the precision of liver resection, and preserving more liver tissue. Optimized anesthesia and meticulous hemostasis are crucial for maintaining stable intraoperative conditions and ensuring adequate blood supply to the liver, which are vital for optimizing the postoperative liver regeneration environment. Furthermore, innovative surgical techniques, such as ALPPS and LVD (liver venous deprivation), increase the liver reserve volume before major hepatectomy.^825,828^ The optimization of embolization materials used in PVE also promotes greater liver growth.^826^ These advancements collectively enhance repair and regeneration.

Lifestyle optimization is a promising intervention for enhancing liver regeneration.^818^ In a randomized controlled trial involving 62 live liver transplant donors, lifestyle modifications significantly increased liver volume regeneration.^819^ The intervention group, which adhered to a specific diet and exercise regimen, demonstrated notable improvements in liver regeneration, with a statistically significant difference compared to the control group. Additionally, this group exhibited higher levels of inflammatory markers, such as TNF-α and IL-6, while showing lower levels of TGF-β, indicating a distinct inflammatory profile during regeneration.^819^

## Conclusions and perspectives

Given the liver’s central position in metabolic equilibrium, as well as the considerable health burdens presented by liver disorders, further exploration of the molecular mechanisms involved in liver repair and regeneration is crucial. The liver repair process involves signaling pathways controlled by multiple cells. These cells not only influence the proliferation of hepatocytes and repair of damaged tissues but also play important regulatory roles in maintaining liver structure, controlling inflammation, and restoring function.

Pathways such as the Wnt/β-catenin, Hippo/YAP, Notch, Hedgehog, TGF-β, PI3K-AKT, TNF-α, and IL-6 signaling pathways involve complicated systems of receptors, signaling molecules, secondary messengers, transcription factors, and effectors that are crucial for normal liver repair and regeneration. A comprehensive analysis of their functions, regulatory processes, and crosstalk mechanisms can considerably expand our knowledge of the molecular mechanisms involved in liver repair and regeneration. Moreover, metabolic reprogramming is intrinsically connected with the signaling pathways that control the repair of the remnant liver. This process involves remodeling in the metabolism of glucose, lipids and amino acids, which provides the necessary energy, materials, and signals to maintain cellular vitality and functionality.

Analyzing the molecular mechanisms of repair and regeneration in several different liver diseases, including AILI, NAFLD/NASH, ALD, viral hepatitis, and age-related conditions, and identifying commonalities and distinct features will contribute to a better understanding of the repair process and the development of targeted and optimized therapeutic interventions. Despite deep preclinical investigations of liver repair and regeneration mechanisms and the proven high potential of clinical trials, safety issues should not be underestimated.

### Limitations and challenges

Although there has been significant progress in identifying the molecular mechanisms of liver repair and regeneration, there are major difficulties in translating these mechanisms into real clinical applications. Among the main problems are inadequate information about the complex interactions of signaling pathways and cellular communication. The influence on one component may have many unintended side effects. Current techniques of modulation do not provide the necessary level of precision. Moreover, variations in expression profiles, as revealed by spatial transcriptomics, point to more complex, localized mechanisms that affect liver regeneration.^756,785^

Metabolic reprogramming is a dynamic process that requires direct and continuous, longitudinal measurements throughout the process of liver repair and regeneration. Most studies present only snapshots of the dynamic at particular time points, which means that their coverage offers only limited insights, failing to capture the full complexity of the process.^786^

### Future directions for enhancing liver repair and regeneration

Future studies should integrate multiomics data, such as genomics, transcriptomics, proteomics, and metabolomics data, to comprehensively map how different signaling pathways interact. This would contribute to identifying potential targets to more precisely regulate liver regeneration processes. Higher resolution and coverage of spatial transcriptomic techniques could be used to identify localized cellular responses that contribute cooperatively to improving overall liver function and regeneration. Future research should also focus on longitudinal studies to track metabolic changes over time instead of at fixed time points to obtain a deeper understanding of metabolic reprogramming during liver repair and regeneration.^786^ Moreover, there is a critical need to develop advanced models that more accurately mimic human liver regeneration, especially in the context of liver disease.^756^ Advances in materials technologies such as organ-on-a-chip and 3D bioprinting could be important for this purpose.^829,830^

Moreover, effectively bridging the gap between scientific research and clinical applications depends on translational research, which calls for interdisciplinary collaborations in fields such as molecular biology, pharmacology, bioengineering, and clinical medicine. Through the development of specific drugs and improvements in drug delivery systems, future research aims to create new therapies that are suitable for widespread clinical use. Looking ahead, by integrating genomic, proteomic, and metabolomic data from patients, therapies can be customized based on specific molecular profiles, significantly increasing the efficacy and safety of regenerative treatments.

This review provides an overview of the molecular mechanisms underlying liver repair and regeneration, connecting fundamental research with clinical applications. In the future, developing targeted therapies that focus on specific signaling pathways and metabolic reprogramming holds potential for enhancing liver regeneration and improving patient outcomes.
